# Scorpion incidents, misidentification cases and possible implications for the final interpretation of results

**DOI:** 10.1186/s40409-016-0075-6

**Published:** 2016-07-02

**Authors:** Wilson R. Lourenço

**Affiliations:** Muséum National d’Histoire Naturelle, Sorbonne Universités, Institut de Systématique, Evolution, Biodiversité (ISYEB), UMR7205-CNRS, MNHN, UPMC, EPHE, CP 53, 57 rue Cuvier, 75005 Paris, France

**Keywords:** Scorpion, Dangerous species, Misidentification, Biased results, Buthidae, *Tityus*

## Abstract

The aim of this contribution is to bring general information on the classification and in particular on the specific identification of scorpion species dangerous to humans. Several generic groups are taken into consideration, but the Neotropical genus *Tityus* C. L. Koch, 1836 is used as a major example. The content of this paper is mostly addressed to non-specialists whose research embraces scorpions in several fields such as venom toxins and public health. Although efforts have been made in the last 20 years to create better links between ‘true scorpion experts’ and non-specialists who use scorpions in their research, such exchanges had never led to a consensus among those different branches of biological and medical research. Consequently, many cases of species misidentification and even more serious errors concerning scorpion classification/identification are often present in the specialized literature. In conclusion, it is suggested here that the frequent cases of misidentification observed in several reports may induce mistakes in the final interpretation of results, leading only to more inefficacity in the treatment of problems caused by infamous scorpion species.

## Background

Scorpions are a rather depauperate group within the class Arachnida with approximately 2,100 known species. They can be classified among the most ancestral arthropods both in origin and body morphology. They first appeared as aquatic organisms during the Silurian (approximately 450 million years ago – MYA) and underwent rather small morphological changes since [1]. Their apparent conservative form led some authors to define them as ‘living fossils’. However, scorpions most certainly developed major biochemical, physiological, behavioral and ecological adaptations that have combined to ensure their continued success over the past 450 million years.

Even if for natural history experts scorpions can be considered fascinating animals, the interest shown by people in general is only connected with their negative reputation of ‘killers of men’. Nevertheless, only a limited number of species, probably less than 50, are actually responsible for serious or lethal incidents. It is true, however, that the interest on scorpion research in many distinct biological fields was generated by the fact that a number of species possess venoms with potent toxins, capable of killing humans. Most deadly species belong to the family Buthidae; though, species belonging to two other families, Scorpionidae and Hemiscorpiidae, also threaten humans.

The group first appeared as aquatic organisms. In their evolutionary history, scorpions almost certainly evolved from Eurypterida (‘water scorpions’) since both groups share several common morphological features. Marine and amphibious scorpions most certainly persisted well into the Carboniferous (359–299 MYA) and some species probably reached the Permian (299–251 MYA) and Triassic (251-200MYA) periods [2, 3]. The first unequivocally terrestrial (air-breathing) scorpion most certainly appeared on land during the late Devonian (416–359 MYA) or early Carboniferous [1, 4]. For a precise description of scorpion evolutionary history and toxin evolution, readers can refer to Lourenço [5].

From the beginning of scorpion studies in the late 18th century and during almost 150 years, researchers focused primarily on descriptive taxonomy, general anatomy and very rudimentary biogeography, followed by medical research on venom biochemistry. Since the 1970s, however, basic research on scorpions expanded greatly to encompass behavior, physiology, ecology evolutionary and reproductive biology. Moreover, a large amount of all the research was based on toxin studies and public health problems caused by dangerous species. New approaches were attempted in this last field of research with the use of more precise methods based on a better knowledge of life history strategies of these organisms [6–9].

In the general mass of recent publications devoted to venoms, toxins and public health problems, it is yet possible to observe major gaps between the most up-to-date information on scorpion identification/classification produced by true scorpion experts and the information diffused by researchers who use these organisms in their own research, but are not aware of their precise classification. For this reason, it is suggested here that the frequent cases of misidentification observed in many reports may induce important mistakes in the final interpretation of results, which can only lead to more inefficacy in the treatment of problems caused by dangerous scorpion species. In this article, I attempt to explain and elucidate a number of common problems with clarity, accuracy and unambiguous communication, hopping that in this way the matter may be accessible to a broad audience.

## How to approach the identification of scorpions

### Techniques for collection

**Fig. 1:**
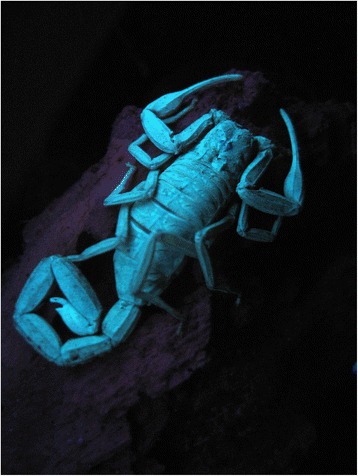
*Tityus brazilae* Lourenço & Eickstedt, 1984; female from Brazil under UV light (copyright by Tiago Porto, reproduced with permission)
- Methods of extraction, such as Berlese, Winkler and Kempson may also be successfully employed to obtain very small species, or juveniles, that inhabit organic soil [13]. These diminutive species, in general with less than 1.5 cm in total length, generally escape from the collection if the other already described methods are used.

As already outlined in some of my previous publications, techniques for collecting and sampling are very important to estimate the composition of the scorpion fauna of a given region [10, 11]. Once the sampling is carried out, the identification process may be attempted by the study of some precise characters. There are four main techniques for the collection of scorpions:

- Overturning stones, rocks and logs beneath which many species can be found. This method, although the most widely used, is time and energy consuming, and the results obtained are generally poor.
- Pitfall traps may be placed in the soil, when fieldwork is expected to last at least for several days, and preferably for some weeks, in the same site. Results obtained by this method can be very satisfactory if the site chosen contains a large population of scorpions. This method is most successful during dry season, but the traps should be checked daily if adequate results are to be obtained.
- Collection at night with the use of a portable ultraviolet light. Because of the presence of riboflavins in their exocuticles, scorpions become fluorescent under ultraviolet light (Fig. 1) and can be easily found [12]. This method is most efficient in open areas such as deserts and savannas; however, it can also be used in rainforests. Generally, results are better in the absence of moonlight. This technique should be used with prudence, since snakes are not fluorescent and can represent a danger in dark nights.

Scorpions are often rather rare and the population densities of many species are low. For this reason, it is usually recommended that specimens should be brought alive into the laboratory for study. They can be maintained in boxes with saturated cotton wool in a dish. The bottom of the box must be covered with a layer of soil of about 2–3 cm in depth, preferably from the region where the scorpion was collected. Food such as spiders or insects (but not ants) should be offered once a week. In many cases, reproduction takes place, thereby providing additional material for study. Once specimens have died they should be preserved in 75 % alcohol [14].

### Taxonomic study and identification

The taxonomy of scorpions presents difficulties comparable with those encountered in most other groups of Arthropods. Before beginning the study of scorpions, it is necessary to be able to recognize all of the morphological features used in taxonomy. Recognition of males, females and juveniles is not always a simple task. For many species, males are bigger, with bulkier or longer pedipalps. This is not, however, a general rule, so another important character to be examined is the size and shape of pectines. Almost without exception, pectines in males are bigger, and overlap in their internal side. Recognition of juveniles is almost impossible for non-specialists. Only good samples can authorize the identification of different instars. It is not in the scope of this contribution to bring all the details about the procedures used in the taxonomic study of scorpions. For more details, readers can refer to Lourenço [5, 10, 13, 14].

## Most relevant scorpion groups in toxin research and public health programs

The great majority of scorpion species related to public health problems belong to the family Buthidae C. L. Koch, 1837. In most cases, the same species are equally used in venom and toxin research. The reasons for this convergence are associated with both common tradition of studying the most infamous species, but also because these are in general the most readily available. Buthids represent the most important family within the order Scorpiones, comprising about 50 % of all the known species. This family contains also a remarkable number of generic groups, but only a few genera are well known by a broad audience (e.g. scholar doing research on venoms and toxins). In fact, many of the buthid genera are extremely rare. Many genera are monotypic or are composed of only 2 to 3 species that can be extremely rare. Consequently, these will never cross the scientific way of most biologists interested in scorpions as tools.

Most infamous species, largely used in venoms and toxins research, but also concerned by public health programs, are globally confined to a limited number of genera. Nevertheless, a great confusion in their classification can yet prevail and the cases of misidentification are rather common. In the next section, I attempt to bring some light over these groups.

### Old World genera

#### Family Buthidae C. L. Koch, 1837

##### The genus *Buthus* Leach, 1815

**Fig. 2:**
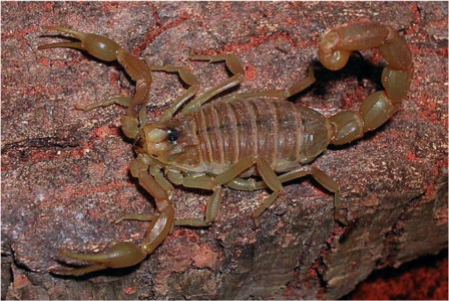
*Buthus occitanus* (Amoreux, 1789); female from France (copyright by Eric Ythier, reproduced with permission)

**Fig. 3:**
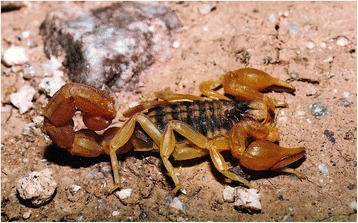
*Buthus albengai* Lourenço, 2003; male from Morocco (copyright by Philippe Geniez, reproduced with permission)

**Fig. 4:**
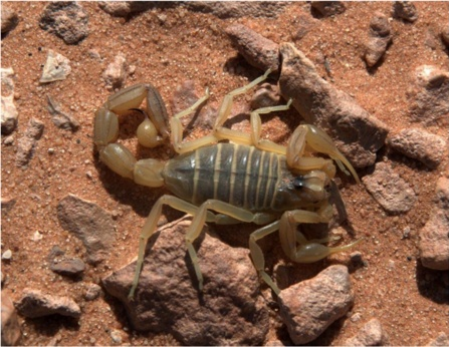
*Buthus saharicus* Sadine, Bissati & Lourenço, 2016; female from Algeria

The genus *Buthus* can be considered as the ‘founder’ of the family Buthidae (Figs. 2, 3, and 4). After its creation by Leach [15], many if not most true buthid scorpions were accommodate in this genus, and this situation persisted over most of the 19th century and even during a large part of the 20th century. Before the creation of *Buthus*, most scorpions were simply placed in the original genus *Scorpio* Linnaeus, 1758. Nevertheless, a great confusion lasted for many decades since numerous species having nothing in common with *Buthus* were also placed in this genus. Even after the creation of other buthid genera, the precise placement of several species remained unclear, and depended on the personal opinion of each author.

It is quite clear, however, that even in present days the taxonomy of the genus *Buthus* remains complex and confusing. In his monograph about North African scorpions, Vachon [16] attempted to establish a better definition of this genus and transferred to other, recently created at that time, genera several species previously included in it. The classification proposed by Vachon [16] for the species of *Buthus*, and in particular for those belonging to the ‘*Buthus occitanus*’ complex of species, remained, however, unsatisfactory, mainly because of the existence of several poorly defined subspecies and even varieties.

Only after the preliminary revision attempted by Lourenço [17], a more precise definition of the species of *Buthus* belonging to the ‘*Buthus occitanus*’ complex was achieved. This situation was followed by the description of several new species and the promotion of subspecies to species rank. Other contributions have also attempted to clarify the taxonomic status of some of *Buthus* species associated with *Buthus atlantis* Pocock [18–20]. The studies on the genus *Buthus* are maintained in a rather positive pace and many other new species are continuously described [21, 22]. If in the monograph published by Vachon [16] only four species of *Buthus* were considered valid, today this number surpasses 30 species and will certainly increase yet.

The species of *Buthus* are distributed throughout Euro-Mediterranean countries, but most of them are widely found from North to Central Africa. Some species are also present in the nearby Middle East. Although the genus *Buthus* is not present in Asia, in many publications devoted to venoms and toxins, authors refer to *Buthus* in India or China. These are clearly cases of misidentifications with the genera *Hottentotta* Birula, 1908 and *Mesobuthus* Vachon, 1950 [23]. Incidents caused by some African species of *Buthus* can be dangerous for young children, but do not reach the same scale of gravity as those caused by other genera of North African buthids.

##### The genus *Androctonus* Ehrenberg, 1828

**Fig. 5:**
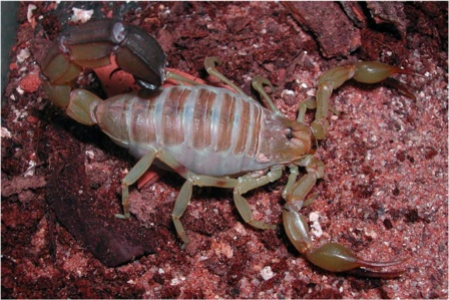
*Androctonus australis* (Linnaeus, 1758); female from Algeria (copyright by Eric Ythier, reproduced with permission)

**Fig. 6:**
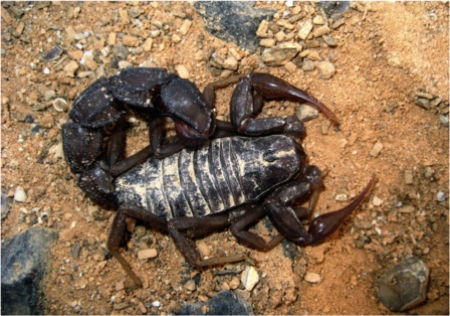
*Androctonus crassicauda* (Olivier, 1807); female from Jordan (copyright by Philippe Geniez, reproduced with permission)

The genus *Androctonus* (Figs. 5 and 6) contains one of the most infamous known species of scorpion, *Androctonus australis*, originally described as *Scorpio australis* Linnaeus, 1758. When the genus *Androctonus* was created by Ehrenberg [24], the reputation of this scorpion group was already well known, since its generic name means ‘killer of men’.

The taxonomy of the genus *Androctonus* Ehrenberg has long remained complex and unclear, with species misplaced in other genera such as *Buthus* or *Prionurus*, 1828, genus now considered as a synonym of *Androctonus*. Vachon [16] in his contribution to the study of North African scorpions attempted to establish a better definition of the genus *Androctonus* and its species. His results were not globally satisfactory, but the number of known species in the genus, between 8 and 10, remained stable until the end of the 20th century, with, however, several poorly defined subspecific populations. After more than half a century of the monographic work of Vachon [16], Lourenço [25] attempted again to characterize the distinct populations of *Androctonus*. It is worth noting that during the entire period between the two contributions, no other authors proposed any major modification on the classification of the species of *Androctonus*. Contributions as the one by Levy and Amitai [26], in their *Fauna Palaestina*, maintained a very conservative position with only minor remarks on some species. With the contribution by Lourenço [25], a few species have been synonymised, some subspecies raised to the rank of species and new species described. After the publication of this first clarification on the taxonomy of *Androctonus*, more new species have been added to the genus [27–33]. Presently, the genus *Androctonus* contains about 20 species, some of which require reinvestigation.

The range of distribution of the genus *Androctonus* is extremely wide, extending, in Africa, from Senegal to East Africa over a line following the 10° N parallel. In Asia, the genus ranges from the nearby Middle East to India, including also countries such as Armenia [34]. *Androctonus* includes large scorpion species with extremely toxic venoms to humans and mammals in general, being the first agent responsible for human incidents and scorpion public health threat in most North African and Middle East countries, causing hundreds of casualties every year.

Misidentifications at species levels are yet very frequent in general publications referring to scorpion incidents, but can equally occur in zoological studies. For example, Asian species are cited as African, or African species are cited as Asian. Besides, incorrect identifications are still often attributed to certain populations. Nevertheless, little by little a better understanding of the precise identity and range of distribution of each population is becoming possible thanks also to studies using molecular approaches (Lourenço, not published).

##### The genus *Leiurus* Ehrenberg, 1828

**Fig. 7:**
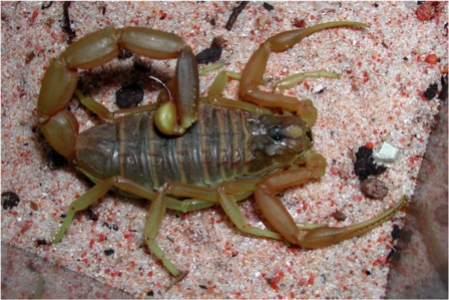
*Leiurus hebraeus* (Birula, 1908); female from Israel (copyright by Eric Ythier, reproduced with permission)

**Fig. 8:**
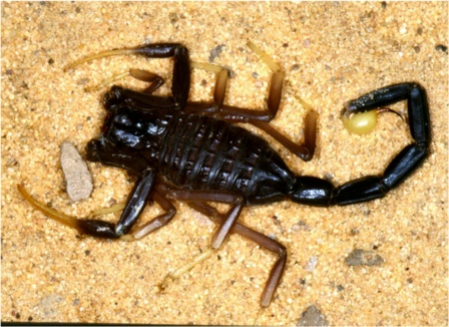
*Leiurus jordanensis* Lourenço, Modry, Amr, 2002; female from Jordan

The genus *Leiurus* (Figs. 7 and 8) was represented over many decades by a single species, *Leiurus quinquestriatus*, containing two subspecies, *L. quinquestriatus quinquestriatus* (Ehrenberg, 1828) and *L. quinquestriatus hebraeus* (Birula, 1908). This scorpion is one of the most common species in desert fauna, in particular in certain regions of Sudan, and especially around Khartoum and Omdurman, as well as in Israel where it was classically represented by the subspecies *L. q. hebraeus* [35–37]. *Leiurus* species secrete one of the most noxious venoms among buthid scorpions in general, and are responsible for extremely serious human incidents. Fortunately the amount of venom produced by an average sting is rather small (0.225 mg) and, consequently, the lives of adult humans are seldom endangered, although the Sudanese population of *Leiurus* is a significant cause of death among young children [38]. Although the incidents caused by *Leiurus* species may be considered as rather severe, these are less frequent, for instance, that those caused by species of *Androctonus*. This is due to the fact that human populations are much less dense in the regions where *Leiurus* species are distributed. Misidentifications of species responsible for the incidents, at least in Africa, are equally less frequent having into account the yet reduced number of known species in this continent.

Because of its infamous reputation as a very dangerous scorpion, the toxins of *L. quinquestriatus* and *L. hebraeus* have been the subject of numerous biochemical studies [39, 40]. The sensory physiology and behavior of *Leiurus quinquestriatus* have been investigated by a number of authors, including Cloudsley-Thompson [35, 36, 38, 41]. Nevertheless, many aspects of the taxonomy of the genus *Leiurus* have long been controversial.

A historical account of the taxonomy of the genus *Leiurus* shows that when the original description of *Androctonus* (*Leiurus*) *quinquestriatus* Ehrenberg, 1828 [24] was provided, this species was originally placed in the genus *Androctonus* and *Leiurus* was considered only as a subgenus of *Androctonus*. This attests the stage of indecision in the classification on the early 19th century. Several subsequent authors, such as Kraepelin [42], regarded *Leiurus* as a synonym of the genus *Buthus* Leach. Finally, Vachon [43] established *Leiurus* as a separate genus with only one species, *Leiurus quinquestriatus*. Vachon [43] was confident that this genus was monotypic, but refrained from revising its intraspecific structure. Two subspecies were considered to be valid by Vachon [43]: *Leiurus quinquestriatus quinquestriatus* (Ehrenberg, 1828) and *Leiurus quinquestriatus hebraeus* (Birula, 1908). Subsequently, the systematic position of *Leiurus quinquestriatus hebraeus* (now recognized as a valid species) has been reviewed by Levy et al. [37], who presented tables that differentiate this subspecies from *L. q. quinquestriatus*.

The position of the two subspecies was considered again by Levy and Amitai [26]. *Leiurus quinquestriatus* has been also the subject of regional investigations. In his study of the scorpions of Saudi Arabia, Vachon [44] mentioned *Leiurus quinquestriatus* without making any reference concerning the subspecies. He also commented on the subspecies *Androctonus quinquestriatus brachycentrus* (Ehrenberg, 1828), suggesting that more material would be necessary for the variability of coloration of metasomal segment five to be defined. Subsequently, Sissom [45] also cited *Leiurus quinquestriatus* in a study of the scorpions of Yemen, but decided not to assign the Yemen population to any particular subspecies.

It was only almost 170 years after the original description of the genus *Leiurus* that Lourenço et al. [46] described a new species, *Leiurus jordanensis* Lourenço, Modry & Amr, 2002 from Jordan. This species was later recorded from Saudi Arabia by Hendrixson [47]. Only a few years after the description of *L. jordanensis*, Lourenço et al. [48] described another new species, but this time from Africa. *Leiurus savanicola* Lourenço, Qi & Cloudsley-Thompson, 2006 was collected is the transitional zone between the Sahel and savanna formations, consequently out of the typical desert formations where *Leiurus* species are commonly found. The new species was found in a burrow under a rock.

In recent years, some other new species have also been described by different authors. In a rather extensive paper, Lowe et al. [49] proposed a full revision of the genus *Leiurus*, but dealing mainly with the populations from the Arabian Peninsula. The status of some old species was revalidated, one recently described species was placed in synonymy, one subspecies was raised to species and four new species were described. This raised the total number of species in the genus *Leiurus* to ten. The characters used by these authors to define the species, as well as the proposed dichotomic key are rather difficult to be used. Nevertheless, one has to agree when these authors stated that *‘like many other scorpion genera,* Leiurus *is comprised of an assemblage of allopatric or parapatric species spread across different regions separated by physiographic barriers, each adapted to local environments and substrates’.* In a very recent publication by Lourenço & Rossi [50], this assumption was once again confirmed, attesting that the African species of *Leiurus* are yet neglected and require intensive further studies. A new species from Somalia, *Leiurus somalicus* Lourenço & Rossi, 2016 was described, raising the number of *Leiurus* species to eleven.

The geographical distribution of the genus *Leiurus* can be summarized as follows: the range of distribution covers Algeria, Chad, Egypt, Ethiopia, Libya, Mali, Niger, Somalia, Sudan and Tunisia in Africa; and Sinai, Iraq, Israel, Jordan, Kuwait, Lebanon, Oman, Qatar, Saudi Arabia, Syria, Turkey, United Arab Emirates and Yemen in Asia. Most African populations certainly correspond to the species *L. quinquestriatus*, whereas those from Asia correspond to *L. hebraeus*, and in small portions to one of the several new species described in the last 15 years. The Isthmus of Suez apparently corresponds to the border between the African species of *Leiurus* and those distributed in the Middle East [26, 37]. The species *Leiurus savanicola*, only known from the Sahel in Cameroon, and *Leiurus somalicus*, found only in the south of Somalia, represent the most southern records on the distribution of the entire genus *Leiurus*.

##### Other genera also involved in incidents and misidentifications: *Hottentotta* Birula, 1908, *Mesobuthus* Vachon, 1950, *Odontobuthus* Vachon, 1950 and *Parabuthus* Pocock, 1890

**Fig. 9:**
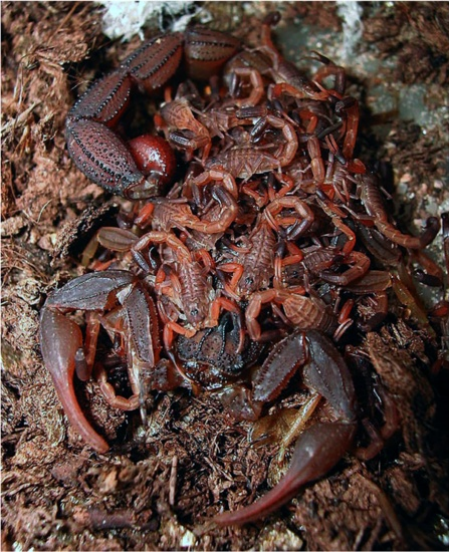
*Hottentotta caboverdensis* Lourenço & Ythier, 2006; female with offspring from Cape Verde (copyright by Eric Ythier, reproduced with permission)

**Fig. 10:**
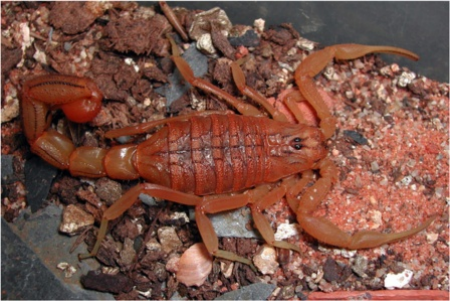
*Mesobuthus tamulus* (Fabricius, 1798); male from India (copyright by Eric Ythier, reproduced with permission)

**Fig. 11:**
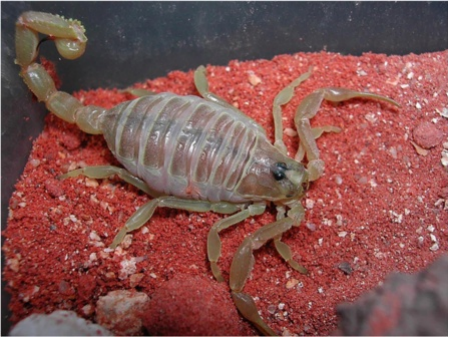
*Odontobuthus odonturus* (Pocock, 1897); pre-adult female from Pakistan (copyright by Eric Ythier, reproduced with permission)

**Fig. 12:**
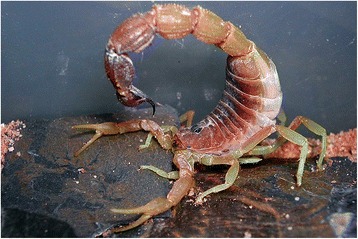
*Parabuthus mossambicensis* (Peters, 1861); female from Mozambique (copyright by Eric Ythier, reproduced with permission)

I will not provide too many details about these buthid genera, largely distributed in Africa, Middle East, Central and Eastern Asia (Figs. 9, 10, 11 and 12). Most of the species included in these genera have been for quite long simply accommodated in the genus *Buthus*. It was only since the monographic work by Vachon [16] that a more accurate subdivision of what was then the genus *Buthus* led to the recognition of approximately ten distinct genera. One of the genera proposed by Vachon [43] was *Buthotus*. This comprised the majority of species in the old subgenus *Hottentotta* Birula. However, Kraepelin [42] had been the first to distinguish a ‘*hottentotta* group’ (species-group) within the genus *Buthus*. Most of the species within it were allied to *Buthus hottentotta* (Fabricius, 1787). Subsequently, Birula [51] created the subgenus *Hottentotta*, but without explaining his motive. Vachon [43] disregarded *Hottentotta* Birula and established a new name, *Buthotus* Vachon, 1949. *Hottentotta* is, however, a valid senior synonym for *Buthotus* and was re-established by Francke [52].

The precise composition of several of these genera and in particular of the four previously cited, which are frequently misidentified with *Buthus*, is not completely clarified. Besides, the unclear position of both *Hottentotta* and *Mesobuthus* species distributed in Asia creates a great confusion in their identification. It seems clear that the genera *Hottentotta* and *Mesobuthus* are far from being uniform, and the used diagnostic characters are very often imprecise. Naturally, the exact clarification of most taxonomic situations is a matter for true experts, nevertheless it can retained that the genus *Buthus* has a distribution in Africa limited to the regions of the Sahara and Sahel. Its presence in the Middle East is rather discrete, with a distribution to East limited to Iraq [53]. Any citation of *Buthus* to Central and Eastern Asia has to be considered as misidentification with the genera *Mesobuthus* or *Hottentotta* [23].

#### Family Scorpionidae Latreille, 1802

##### The genus *Scorpio* Linnaeus, 1758

**Fig. 13:**
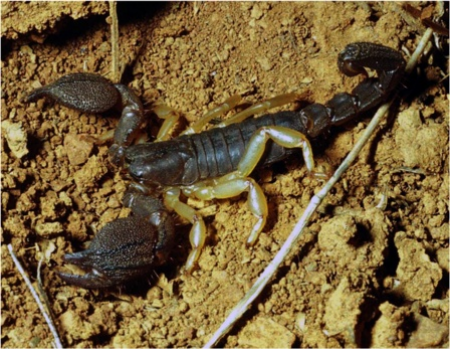
*Scorpio maurus* Linnaeus, 1758; male from Tunisia

The genus *Scorpio* (Fig. 13) with the species *Scorpio maurus* Linnaeus, 1758 can be considered as the original nomination defined in the zoological nomenclature by Linnaeus [54]. For a long time, and in particular since the conservative classification proposed by Vachon [16], only one widespread and highly polytypic species was recognized, *S. maurus*. It seems obvious, however, that this unclear taxonomic situation could not last, specially having into account that *Scorpio* species possess toxic venoms with a hemolytic action [55]. Therefore, a precise clarification of the specific status of different populations was necessary. Lourenço [56] and Lourenço and Cloudsley-Thompson [57, 58] reinvestigated the taxonomic position of several species of *Scorpio* based on a number of characters already defined by Vachon [16] and confirmed that these were valid species. Using this approach, eight forms or subspecies were raised to the rank of species. Subsequently, new species were also described, e.g., *Scorpio savanicola* Lourenço, 2009; *Scorpio sudanensis* Lourenço & Cloudsley-Thompson, 2009; *Scorpio niger* Lourenço & Cloudsley-Thompson, 2012; and more recently *Scorpio tassili* Lourenço & Rossi, 2016 from the Hoggar Mountains in Algeria. With these new discoveries, species of *Scorpio* were reported from beyond the Saharan region for the first time [59].

The initial decision taken by Lourenço [56] of raising several forms or subspecies of *Scorpio* to the rank of species was received with scepticism by a number of authors (several reactions *in litt*.). Nevertheless, in recent publication Talal et al. [60], using more sophisticated molecular methods, followed the same analysis, previously started by Lourenço [56], and concluded that the raising of some Middle East subspecies of *Scorpio* to the rank of species was necessary. Previous analysis on some *Scorpio* species by Lourenço and Cloudsley-Thompson [58] suggested that those characterized by a pale coloration – e. g. *Scorpio tunetanus* Birula, 1910 (now *Scorpio punicus* Fet, 2000); *Scorpio occidentalis* Werner, 1936; *Scorpio savanicola* Lourenço, 2009; and *Scorpio niger* Lourenço & Cloudsley-Thompson, 2012 – most certainly corresponded to possible members of a single group that originated from a common ancestor, but today they occupy distinct regions of distribution. This position was corroborated by the recent discovery and description of *S. tassili* by Lourenço & Rossi [59]. The range of distribution of *S. punicus* appears to be limited to the high plateaus of Tunisia and north of Algeria [16, 61], whereas the other species of this group are distributed much further to the south, in the Sahel region.

The precise taxonomic situation of all *Scorpio* populations is not yet fully clarified. The species of this genus have a widespread distribution, ranging from Senegal to Sudan and Egypt in Africa and covering most regions of Middle East.

#### Family Hemiscorpiidae Pocock, 1893

##### The genus *Hemiscorpius* Peters, 1861

**Fig. 14:**
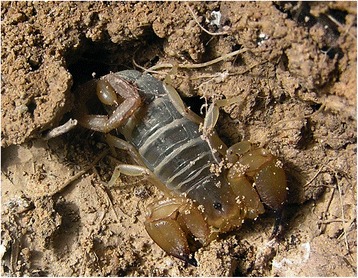
*Hemiscorpius lepturus* Peters, 1861; female from Iran (copyright by Philippe Geniez, reproduced with permission)

Scorpions of the family Hemiscorpiidae Pocock, 1893 and of the genus *Hemiscorpius* Peters, 1861 (Fig. 14) have been the subject of few studies, aside from the original descriptions. Two recent noticeable exceptions are the publications of Monod and Lourenço [62] and Lowe [63]. These focused mainly on the Middle East species, in particular those from Iran and Oman. As outlined by Monod and Lourenço [62], among the species known to occur in Iran, *Hemiscorpius gaillard* (Vachon, 1974), *Hemiscorpius persicus* Birula, 1903 and *Hemiscorpius lepturus* Peters, 1861, the last one is of particular medical interest. In fact, *H. lepturus* is a potentially lethal species that is responsible for significant scorpionism problems in the southern provinces of Iran. *H. lepturus* venom is highly cytotoxic with hemolytic action and may cause severe wounds, skin inflammations and deep dermonecrotic slow-healing ulcers and blisters, which results in important scars. Other severe complications may also be observed such as serious haemolysis, internal hemorrhages, renal failure and even death [62, 64, 65].

Besides the presence of the family Hemiscorpiidae in the Middle East, some species are also found in Eastern Africa, Somalia and Egypt [66, 67]. Nevertheless, the Iranian *Hemiscorpius* of the *H. lepturus* group are very divergent from the African species of this genus. The African species do not show the highly derived features presented by the Iranian one, such as a conspicuous sexual dimorphism and highly cytotoxic venom [62].

### New World genera

#### Family Buthidae C. L. Koch, 1837

##### The genus *Centruroides* Marx 1890

**Fig. 15:**
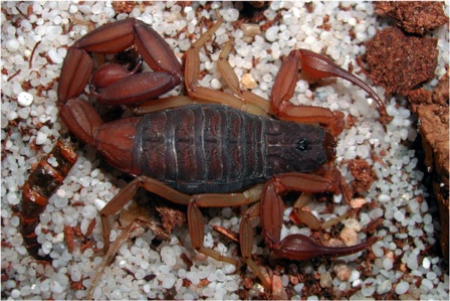
*Centruroides ochraceus* (Pocock, 1898); female from Mexico (copyright by Eric Ythier, reproduced with permission)

**Fig. 16:**
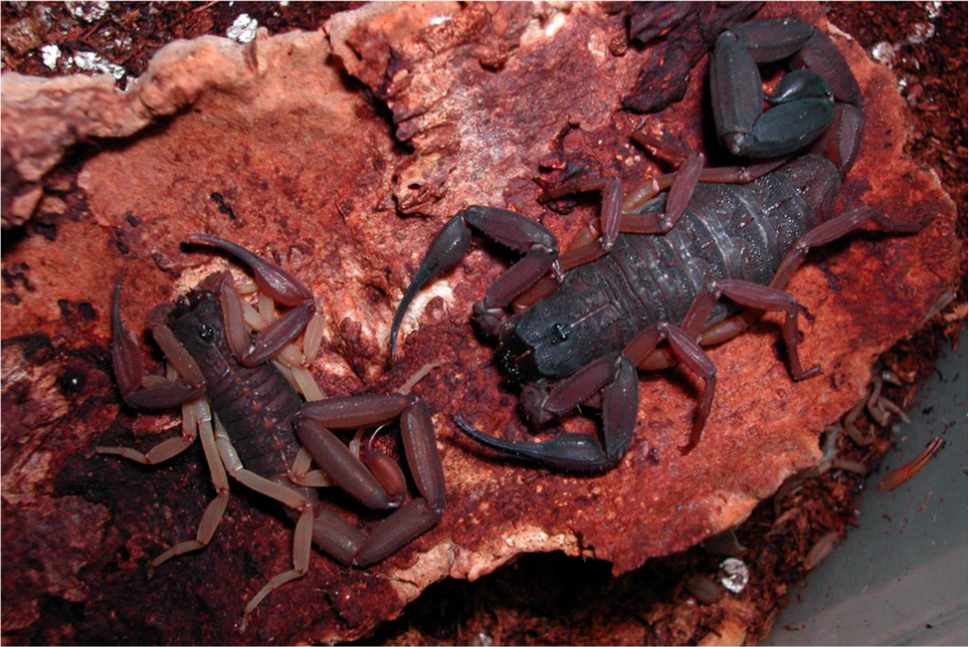
*Centruroides gracilis* (Latreille, 1804); male and female from Colombia (copyright by Eric Ythier, reproduced with permission)

*Centruroides* (Figs. 15 and 16) is the only largely speciose buthid genus present in Central and North Americas. *Centruroides* can be considered a large genus with 70 to 80 described species that are distributed from southern United States to northern South America and the Caribbean islands. Several species are based heavily on color and morphometrics, both of which have been shown to exhibit great intraspecific variation. It is likely that several species, in particular many of the recently described ones from the Greater Antilles, will prove to be invalid. On the other hand, other species will be proven to represent complexes of sibling species rather than single species.

The center of distribution of *Centruroides* is probably Central America [68]. The species of this genus are distributed in a great array of distinct habitats, ranging from arid deserts to rainforests. Several species of *Centruroides*, in particular among those distributed in Mexico, possess venoms potentially lethal to humans and constitute a very important health problem. In fact, the number of incidents by scorpion stings in Mexico is from far the most important in the world. The total number of incidents is largely more important than those of any other region concerned by scorpion incidents in the world. A few fossil registrations from Miocene amber attest that this group was already present in the region since the middle of Cenozoic times. This group of scorpions deserves to be treated in detail, but this is out of the scope of the present contribution.

##### The genus *Rhopalurus* Thorell, 1876

**Fig. 17:**
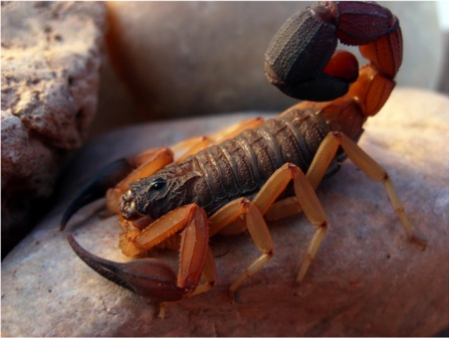
*Rhopalurus agamemnon* (C. L. Koch, 1839); female from Brazil (copyright by Tiago Porto, reproduced with permission)

**Fig. 18:**
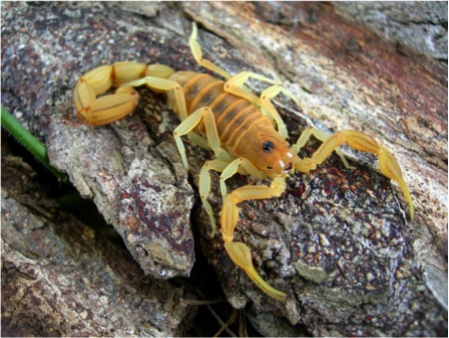
*Rhopalurus rochai* Borelli, 1910; female from Brazil (copyright by Tiago Porto, reproduced with permission)

The genus *Rhopalurus* (Figs. 17 and 18) is a typically Neotropical element distributed mainly in South America, with also some disrupted distributed species in the Greater Antilles. Several species included in this genus are of great general size, reaching up to 12 cm in total length. Nevertheless, most species do not appear as having a major medical importance. Some species, however, seem to be responsible for a number of incidents, mainly in the Amazon region [69]. As already outlined in a recent paper by Lourenço [70], except for most of the Caribbean species of *Rhopalurus*, those inhabiting South America are typical elements of open vegetation formations. The core area of distribution of this genus in South America is composed by savanna-like formations (Cerrado) and semi-arid vegetation (Caatinga) of central and northeastern regions of Brazil. One species is also known from a single enclave in a savanna formation inside oriental Amazonia, and *a priori* a few other species and subspecies are known from the savannas of the Guayana region [71]. Finally, two species are distributed in Venezuela and Colombia respectively in the Llanos of Orinoco and Llanos of the Magdalena [72–74]. In recent studies, the composition and the validity of the geographic and ecological patterns of several species have been discussed and questioned [74–76]. For this reason, the biogeographic situation of this genus was re-opened and new insights in relation to the species distributed in the northern South American savanna formations have been largely discussed [70].

The biogeographic pattern presented by the genus *Rhopalurus* constitutes one good example of a group showing a discontinuous distribution. This type of pattern can be observed in two different cases, among scorpions exclusively adapted to savannas or to rainforests. These examples have an important relationship with species endemic to islands of savanna in Amazonian and Guayanian enclaves [71], but also to those isolated in forest islands inside open vegetation formations (Caatinga, Cerrado and Campos). The endemic populations isolated inside savanna islands provide good evidence to support the hypothesis of past connections between the savannas of Central Brazil and the savanna enclaves in Amazon and Guayana regions. When forest cover was reduced, open vegetation formations probably coalesced during past dry periods which took place at the end of Cenozoic and during Pleistocene times [77, 78].

Scorpion patterns of distribution represent good examples that support this hypothesis. *Rhopalurus* most certainly exhibited a continuous distribution during Pleistocene dry periods and the current disrupted distribution is a possible consequence of the reestablishment of rainforest over the regions which previously served as corridors [70, 72, 79]. Evidence for this presumed palaeodistribution was provided with the discovery and description of the species *Rhopalurus amazonicus* Lourenço, 1986 endemic to a savanna enclave in the region of Alter do Chão (state of Pará, Brazil) and completely isolated within oriental Amazon forest [80]. A careful analysis of *Rhopalurus amazonicus* placed it in a position close to *Rhopalurus acromelas* Lutz & Mello, 1922 that is found in central Brazil, but has a range of distribution nearby the area of transition between Cerrados and Amazon forest.

Since the revision of the genus by Lourenço [81], followed by the reanalysis of its distribution [72], very few new species have been described for South America. Nevertheless, all new discoveries confirmed the biogeographic pattern of distribution already observed for the genus.

The taxonomic status of the species distributed in the northern South American savanna formations always proved to be difficult to be defined. This situation is associated with the fact that these savannas are composed of several isolated fragments that probably coalesced during past dry periods. The most recent of these events can be dated only from 18.000 to 13.000 years before present (BP) [77]. This very recent isolation led to a minor process of speciation and differentiation and, as consequence, the populations now found in several isolate fragments of savannas show very little morphological differences. In face of the observed patterns of distribution and differentiation, it becomes rather difficult to be sure about the true taxonomic status of some isolate populations. Consequently, one question can be addressed: are these populations true species, subspecies or simply local morphs belonging to large polymorphic populations?

The recent analysis of the species of *Rhopalurus* distributed in the savannas of northern South American clearly showed the existence of two distinct lineages [70]. One represented by *R. laticauda* Thorell, 1876 with three associated forms (species or subspecies), and the other represented by *R. pintoi* Mello-Leitão, 1932 with possibly two associated forms. In both cases, the forms within each of the two lineages present only weak morphological differences, demonstrating a recent and only minor process of differentiation. This pattern of both distribution and differentiation is directly associated with more or less recent palaeoclimatic vicissitudes that took place in tropical South America during the Pleistocene [77, 78]. During dry periods, most of the South American savannas and/or open vegetation formations coalesced to form continuous areas of distribution for species exclusively adapted to these formations such as those belonging to the genus *Rhopalurus*. During wets periods, such as the present one, these same savanna formations undergo a process of fragmentation with a subsequent isolation of small populations.

The consequences on the process of speciation during the subsequent wet/dry/wet periods is difficult to measure, but probably was rather weak on groups such as scorpions with long term reproduction process and a low number of generations when compared to other zoological groups such as insects [79, 82]. It is therefore rather difficult to assign a precise status to the forms within the two well-established lineages. Are we in face of species, subspecies or only morphs of a large polymorphic species? If, at least for some of these forms, their specific condition could be demonstrated in association with a clear allopatric distribution of the populations, than the condition of superspecies sensu Mayr [83] could be applied to each of the two lineages. The species within each lineage would be represented by allopatric, parapatric our weakly sympatric groups, really or potentially intersterile in nature [84]. Each of these species could than be defined as a prospecies in the sense of Birula [85].

##### The genus *Tityus* C. L. Koch, 1836

**Fig. 19:**
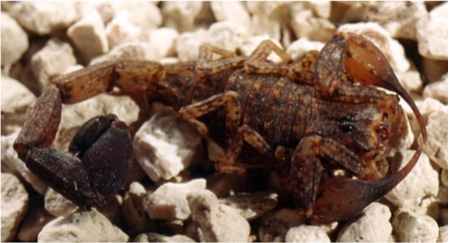
*Tityus* (*Archaeotityus*) *silvestris* Pocock, 1897; male from Brazil

**Fig. 20:**
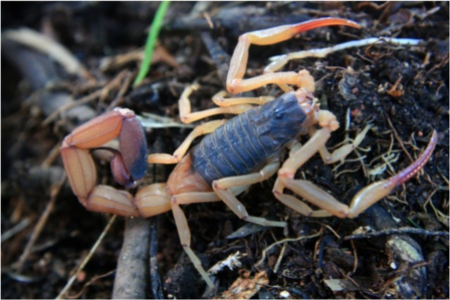
*Tityus* (*Tityus*) *melici* Lourenço, 2003; male from Brazil (copyright by Tiago Porto, reproduced with permission)

**Fig. 21:**
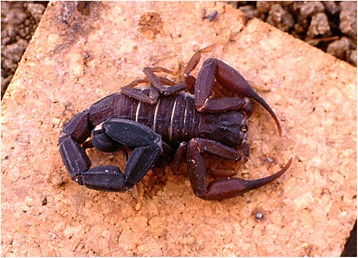
*Tityus* (*Atreus*) *insignis* (Pocock, 1889); male from the island of Saint Lucia

**Fig. 22:**
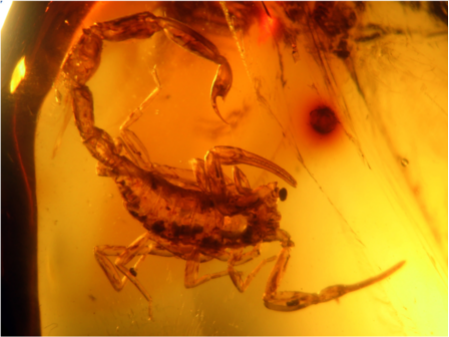
*Tityus* (*Brazilotityus*) *hartkorni* Lourenço, 2009; male in Dominican Amber from the island of Hispaniola; Miocene period

Because of the number of described species, which now overpass 220, the genus *Tityus* (Figs. 19, 20, 21 and 22) is considered the most outstanding among all the scorpion genera. Besides, it is clear that this great number of species cannot be regarded as final, even if some recently described species do require reinvestigation. In account of the importance of the genus *Tityus*, its taxonomical history remains important. For this reason, I propose once again here, a summary of this complex history which was already outlined in previous publications [86, 87].

The genus *Tityus* was first created by C. L. Koch in 1836 [88] having as type species, by monotypy, *Scorpio bahiensis* Perty, 1833 [= *Tityus bahiensis* (Perty, 1833)]. This means that when Perty [89], in 1833, described the species *Scorpio bahiensis*, he placed it in the old genus *Scorpio* Linnaeus, 1758. The type locality indicated by Perty [89] for *Scorpio bahiensis* [= *Tityus bahiensis* (Perty, 1833)] was “*Habitat Prope Bahiam*”, presumably in Brazil since the material he studied was originally collected during the early 19th century expedition performed in this country by J. B. von Spix and C. F. Ph. von Martius. This locality remains, however, extremely imprecise [87].

Since the creation of the genus *Tityus*, a large number of new described species were placed in it. Consequently, very early revisions concerning the status of the different species were required. A pioneer work is the one produced by Kraepelin [90] in 1899, in his *Das Tierreich*. His work was important because it was done in the context of a world revision of all scorpions known to that date. Although this contribution was extremely relevant at that time, covering all the 34 known species of *Tityus*, it could not be final. In fact, for several of these species Kraepelin [90] could only count on very limited samples what invalidated any analysis of the stability or variability of the taxonomic characters used by him.

Subsequently to Kraepelin’s [90] contribution, new species and subspecies were described in an important pace, leading to the need of new revisions. Besides, having into account the increasing complexity of the genus *Tityus*, several authors suggested the need to divide it in several groups of species, more or less ‘natural’. In 1911, Kraepelin [91] had already stated that the genus *Tityus* was the most complex among the American scorpions, because of its large number of species and the difficulties to find useful characters allowing the definition of natural groups. He attempted himself to create three groups of species using the pigmentation patterns: species of large size with blackish-brown pigmentation (group *cambridgei*), species of moderate size with dark longitudinal stripes (group *bolivianus*) and species of small size with a variegated pigmentation (group *columbianus*). Although he had observed a degree of variation in the pigmentation patterns and reached to the conclusion that this character was not entirely satisfactory, he stated, at the same time, that he could not find other characters!

Following the work of Kraepelin, other authors attempted to revise the genus *Tityus*. In 1924, Mello-Campos [92] proposed an identification key to the species, without, however, any division of the genus in natural groups of species. More solid attempts to divide the genus into groups of species finally came in the publications by Mello-Leitão [93, 94] and particularly in his monographic work on all South American scorpions published in 1945. In this study, Mello-Leitão [95] offered a synthesis of all his previous results and divided the genus *Tityus* into 15 ‘Formenkreise’ (what literally means ‘circles of morphs’ or natural groups). After 1945 and until 1975, a number of papers on the genus *Tityus* have been published, but no changes to this classification was proposed.

The matter concerning groups of species – or sometimes complexes – within *Tityus* restarted to be discussed by the end of the 1970s, in particular by Lourenço [96–100], Lourenço and Maury [101], and Lourenço and Eickestedt [102]. Nevertheless, any attempt to proceed to a global revision of the genus proved to be extremely complicate, mainly because of the constant increase in the number of new species (e.g. Lourenço [103–105]). A major attention was than dedicated to the interpretation of the observed patterns of distribution and differentiation of several groups of species [106–109]. At the same time, the number of new described species was constantly growing (e.g. Gonzales-Sponga [110–114], Lourenço and Ythier [115]). Consequently, the number of described species of *Tityus* nowadays overpasses 220 and it is far from being final.

By the early 2000s, the issue of the division of the genus *Tityus* into groups of species was debated again. To a certain degree, Lourenço [10] restored the propositions made by Kraepelin [91] by retaining three major groups of species, based mainly on pigmentation patterns, but also some morphological characteristics. Each of these groups was named after the oldest species belonging to them, reaching the following results: species group *Tityus clathratus*, species group *Tityus bahiensis* and species group *Tityus asthenes*. Two other new insights were equally proposed in this period. The first was the description of a new genus *Caribetityus* Lourenço for the *Tityus* species found in the Greater Antilles [116]; a decision not always accepted by other authors [117]. The second one was the proposition of a new species group for some particular species of *Tityus* found in the Amazon canopy [118].

In the face of all these novelties and polemics among several authors, a division of the genus *Tityus* in several subgenera was finally decided [86]. The objective was to bring further stabilization to the classification of the group. The genus *Tityus* was than divided into five subgenera. Three of them were based on already available genus-group names. Two new subgenera were created: *Archaeotityus* Lourenço to accommodate the species previously associated to the *Tityus clathratus* group, and *Brazilotityus* Lourenço in which Amazonian canopy species were accommodated. The composition of the genus *Tityus* in five subgenera remains as follow: *Archaeotityus* Lourenço, 2006 comprising the *Tityus clathratus* group of species. *Tityus* C. L. Koch, 1836 comprising the *Tityus bahiensis* group of species. *Atreus* Gervais, 1843 comprising the *Tityus asthenes* group of species. *Caribetityus* Lourenço, 1999 comprising several *Tityus* species of the Greater Antilles. *Brazilotityus* Lourenço, 2006 comprising the *Tityus adisi* group of species. These decisions concerning the composition of the genus *Tityus* are still a matter of polemics among certain authors and are rejected by others. Nevertheless, in account of the complexity of this group of scorpions, this decision appeared as fundamental.

Among the enormous array of known *Tityus* species, only a minor number is generally the subject of attention from people managing problems of scorpionism. In the particular case of Brazil, only two/three species of *Tityus* call the attention of professionals working on public health problems. Many subjective arguments are often proposed to explain the causes and dimension of scorpionism; however, precise ecological aspects are frequently neglected. It is quite true that ecological studies on Brazilian and South American scorpions are extremely scarce. Ecological studies using scorpions as models only began in recent years, and most studies were carried out on species from North American deserts [119]. Deserts are globally open formations and this makes scorpion observation rather easy during the night, with the use of ultra-violet lamps. Ecological observations in savannas and in particular rainforest formations, which are common in South America, are more complicate, limiting therefore the number of studies. The combination of these factors has contributed to the present situation regarding ecological research on South American *Tityus*.

However, there are a few exceptions. Ecological studies have been conducted on species inhabiting the Cerrado; one example is *Tityus fasciolatus* Pessôa, 1935, and some data is now available for species living in modified environments such as *Tityus serrulatus* Lutz & Mello, 1922 and *Tityus bahiensis* Perty [10]. Results on the ecology of scorpions should always be interpreted in the light of evolutionary ecology. This approach can have an important impact on studies of biogeography, but also in the understanding of the evolution of the phenomenon of scorpionism. According to the model proposed by Pianka [120], scorpions should be one of the exceptions to the dichotomy proposed by him. Most of the traits of scorpions, with their K-selected strategies, resemble those of vertebrates rather than those of invertebrates [14]. Even if some scorpions are opportunistic species, the majority can be defined as equilibrium species. Consequently, it is reasonable to accept the existence of different evolutionary gradients inside scorpion species in general.

Environmental conditions, stable and predictable or unstable and unpredictable, play a major role in evolution of life history strategies. Some precise cases of secondary succession among *Tityus* populations in South America and Brazil in particular can be suggested. These cases must be considered both in relation to historical factors, such as palaeoclimatic fluctuations, and to ecological factors, such as natural disasters or human activity on the environment. South America and Brazil are favourable regions for this type of discussions since several *Tityus* populations are presently well known and data on historical events that took place during Cenozoic and Pleistocene are rather abundant [79, 82, 107, 121].

Density of scorpion populations are extremely variable, but most species maintain populations with very low densities. Exceptions are found in some desert species, and among some populations that live in disturbed environments where the niche left vacant by equilibrium species has been entirely occupied by one or two opportunistic species [119]. In this last case, populations may present demographic ‘explosions’ that are characteristic of species with density independent regulations. This phenomenon can be observed in some *Tityus* populations, in particular in Brazil. *Tityus serrulatus* and even *Tityus bahiensis* have invaded many towns and cities in the southeast and central regions of the country. In these regions, collection of several thousands of scorpions can be achieved in only a few days.

The patterns of distribution presented by the natural populations of *Tityus* species in South America are complex, but seem to be globally correlated to the major natural morphoclimatical regions of the continent. As already outlined by Lourenço [79], a correct biogeographical view of any region, especially at the continental scale, requires a precise knowledge of its morphoclimatical subdivisions, i.e. the sub-regions where specific conditions of climate and soil can be defined, having as consequence a particular type of vegetation. The heterogeneity of tropical South American vegetation can be described by major type formations based mainly on physiognomic and ecological conditions of climate and soil as discussed by Eiten [122] and Ab’Saber [77]. In the particular case of the species of *Tityus*, three major vegetation formations are recognized. First, the rainforests or wet forests (e.g., the Amazon region, Guayana forest, Pacific Colombian-Ecuadorian forest, Brazilian Atlantic forest), where yearly rainfall varies from 1500 mm to 4000 mm as in Amazonia and Guayana, rising to 9000 mm in the Pacific Colombian-Ecuadorien forest. Secondly, open vegetations, such as dry formations in Chaco and Brazilian northeastern Caatinga, where the yearly average rainfall is 300 to 1000 mm with 7 to 10 months of strong dry season. Finally, non-xerophytic formations as Cerrados in central Brazil, Gran Sabana and Llanos, where the average temperature is 20-26 °C, with a few frosts in winter occurring at the southern edge, and with a average yearly rainfall of 750 to 2000 mm with a moderately strong dry season of 4 to 5 months. Transitional regions (e.g., Chaco/Amazon and Cerrados/Amazon) where intermediate climate conditions can be observed [122]. These major types of vegetation are not uniform and several gradients may occur within each. In fact, the very important diversity observed in tropical South America can be explained, in a large proportion, by this very important heterogeneity.

**Fig. 23:**
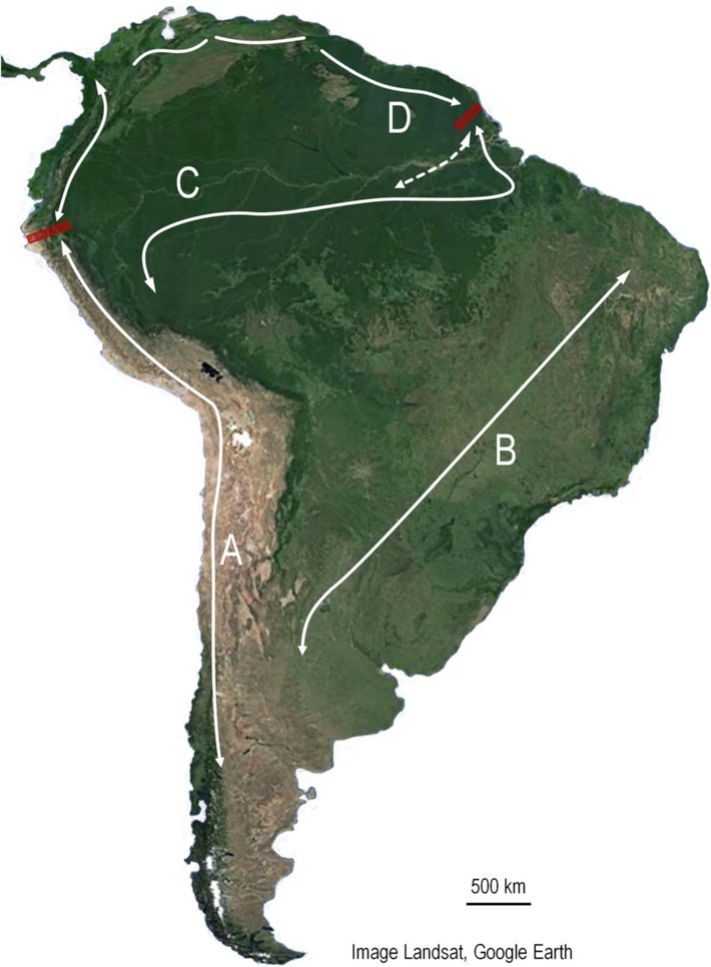
Major corridors of distribution for South American scorpions

Following the definition of the morphoclimatical regions of South America, a number of natural corridors of distribution have been defined for the scorpion fauna since the early 1980s [123]. Because the genus *Tityus* is present in almost all geographical regions and types of vegetation in South America, these corridors can globally be applied to its different populations. The corridors or passages were defined in the sense proposed by Goodnight and Goodnight [124] (Fig. 23).

Corridor A is typically transandine and extends from southern regions of South America (Chile and Argentina) up to the Panama in the north. A zone of transition (or rupture) of some species is observed on the boundary between Peru and Ecuador. This biogeographical barrier is probably associated with the transition from the Pacific coastal rainforest to the Pacific coastal desert. Most typical elements of the transandine region belong to non-buthid familles. The species of *Tityus* (Buthidae) are globally present in the northern part of this region [105, 125].

Corridor B is represented by open vegetation formations that occupy the diagonal from the Monte of Argentina to the Brazilian Caatingas. Typical elements belonging to buthids and non-buthids are present in this corridor. The center of origin for the elements of the genus *Tityus* could be in Brazilian Highlands since their possible centers of dispersion may correspond to the Brazilian shield [126].

**Fig. 24:**
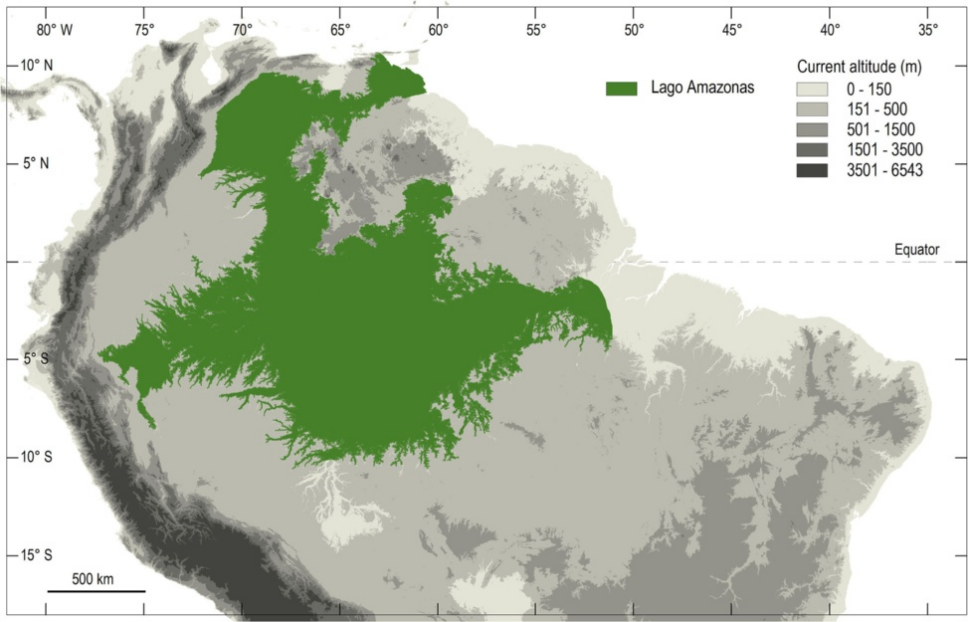
A hypothesized reconstruction of Lago Amazonas with a northern outlet through the Orinoco Valley (modified from Frailey et al. [128])

Corridors C and D occur respectively over the Amazon and Guayana regions; several secondary corridors connect these two parapatric regions. One zone of transition (or rupture) is observed on the boundary between Surinam and French Guiana. This zone probably corresponds to an eco-barrier [127]. Another important zone of rupture occurs in the region of the Orinoco’s delta in Venezuela. A possible explanation for this observed pattern is the hypothesis that central Amazonia was possibly covered by a large inland lake called ‘Lago Amazonas’ [128] in the late Pleistocene and early Holocene (Fig. 24). This lake probably had an outlet through the Orinoco delta. The presence of an important lake certainly acted as a main barrier and produced the present zone of rupture. A number of examples may illustrate precise ecological/biogeographical situations observed in different morphoclimatical regions.

Two species of *Tityus*, with phylogenetic relationship, can show quite different ecological requirements. These are the cases of *Tityus fasciolatus* from central Brazil and *Tityus bahiensis* distributed from southeastern Brazil to Paraguay and north of Argentina. The savannicolous scorpion *Tityus fasciolatus* is a typical equilibrium species that inhabits a stable and predictable environment. The savannas of the Brazilian Highlands are metaclimax formations that are both stable and predictable. These savanna formations are marked by two major seasons: the wet rainy season, from September to April, and a very dry season that lasts from May to August. *Tityus fasciolatus* is termitophilous, sheltering exclusively in the mounds of *Armitermes euamignathus* Silvestri. Its gestation period ranges from 2.5 to 3.5 months and, as in other *Tityus* species, females require only one insemination to produce multiple broods (1 to 5) during the more favourable wet season. As soon as the dry season begins, inseminated females develop reproductive diapause and gestation is blocked until the return of the next wet season, six months later. Diapause is characteristic of equilibrium species. By avoiding parturition during the dry, unfavourable season, immature forms are spared two selective ecological pressures: prey scarcity and the action of savannas fires that are quite common during the dry season. Unlike adults, which shelter exclusively in termite mounds, immature *T. fasciolatus* are errant and often hide under bark. Therefore, they are more exposed to fire [129, 130].

A sibling species of *T. fasciolatus*, *Tityus charreyroni* Vellard, 1932 is equally a savannicolous element but both species occupy different and quite specific microhabitats in the savannas. *T. fasciolatus* is an exclusively termitophilous scorpion while *T. charreyroni* lives under stones in a region western of that occupied by *T. fasciolatus*. The area and range of distribution of each population depends upon the existence and distribution of their specific microhabitat [130, 131]. The construction of termite mounds is associated with particular grasses, which constitutes the specific food for termites [132]. Variation in the soil gradient leads to variation of the covering vegetation. When grasses are no longer present, both termite mounds and *T. fasciolatus* disappear. Moreover, in areas where termite mounds are common, stones are rare, thus making the habitat unfavourable for *T. charreyroni*.

The ecological requirements of *Tityus bahiensis* are much less specific. This species most certainly also evolved in primary savanna formations, but these original savannas inhabited by *T. bahiensis* were destroyed or modified by human activities very early in the history of South America colonization. This species was, however, capable of a considerable degree of adaptation to such modified zones and is found today inside cities and small towns where it colonizes parks, gardens and cemeteries. In contrast, *T. fasciolatus* knew an important regression in the original area of distribution when this one also suffered with human activities. The great ecological plasticity and capacity of colonization of *T. bahiensis* is, though, less marked than that of other *Tityus* species such as *Tityus serrulatus*.

*Tityus serrulatus* Lutz and Mello, 1922 is well known as the most infamous *Tityus* species in Brazil and certainly also among all the known species of *Tityus*. The species was described only in 1922 by Lutz and Mello [133] in a short note devoid of any illustrations. The authors indicated only Belo Horizonte as the type locality and, by that time, it seems that the species was a common one in urban environments. Today, the Brazilian yellow scorpion is well known, even by a large public, and its geographic distribution has expanded considerably since its description. For further details on the history of this species, readers may refer to Lourenço [87]. It is also of general consensus that this species poses exceptional risks to public health due to its rapid expansion in urban areas, rapid proliferation and great toxicity. In fact, all these ecological and biological parameters are linked to its unique modality of reproduction by parthenogenesis. Actually, one point remained enigmatic during several years: the absence of males from all known populations. This question was finally answered by Matthiesen [134], who first demonstrated that this species could reproduce by parthenogenesis. This phenomenon was later demonstrated in a few other species of scorpions, but remains rare [135]. *Tityus serrulatus* was usually considered an obligate parthenogenetic species, but some isolated bisexual populations have finally been detected [136, 137]. However, bisexual populations appear to be extremely marginal within the present geographic distribution of the species, suggesting a possible elimination from most, if not all, of the unpredictable environments occupied by *T. serrulatus* [6, 9].

Several authors have noted that parthenogenetic animals have a tendency to appear in environments that are different from those inhabited by their bisexual relatives [138–142]. Parthenogenetic species also possess higher colonizing and dispersal potential than sexual species and *Tityus serrulatus* conforms to this prediction. Only a few hundreds years ago, this species probably occupied a restricted area in the state of Minas Gerais, but is now widely distributed over a large region of south-eastern and central Brazil and can also reach Argentina and Bolivia via Paraguay and the Brazilian state of Rondonia [7, 9]. Its geographical expansion is undoubtedly due to its introduction into newly created cities and towns by human agency. Newly established human communities may, therefore, be invaded within a few years after their foundation, although the surrounding natural areas (savannas/Cerrados) may be virtually devoid of such species.

The creation of new habitats suitable for colonization by *T. serrulatus* may be compared with natural clearings in dense primary forest [143]. The Cerrados of the Brazilian Highlands may represent such forests and the new towns may be comparable to the clearings. In both cases, the new environments represent insular-type habitats that are now known to favor the establishment of parthenogenetic populations [141]. The new towns are, in many cases, separated by several hundred kilometres. Consequently, the area between them remains almost pristine, representing a formidable barrier to colonization. When parthenogenetic scorpions are transported by anthropogenic agents, such as road or rail, the process of colonization is greatly accelerated. It is greatly facilitated by the higher prolificacy and superior colonizing ability inherent in the parthenogenetic mode of reproduction, which favors the colonization of remote and unoccupied territory [141]. However, such territory does not need to be different, or a disclimax, as is currently believed by many authors; it should be merely unoccupied [144]. In the case of parthenogenetic cave crickets, Hubble and Norton [144] found that the environments of the sexual and unisexual forms are equable, humid, undisturbed and highly predictable. The essential factor for the establishment of parthenogenesis in this case is that remote and uninhabited caves must be colonized by sweepstake dispersal in which the chances for success are very small for any individual and overwhelmingly against the simultaneous arrival of both sexes.

**Fig. 25:**
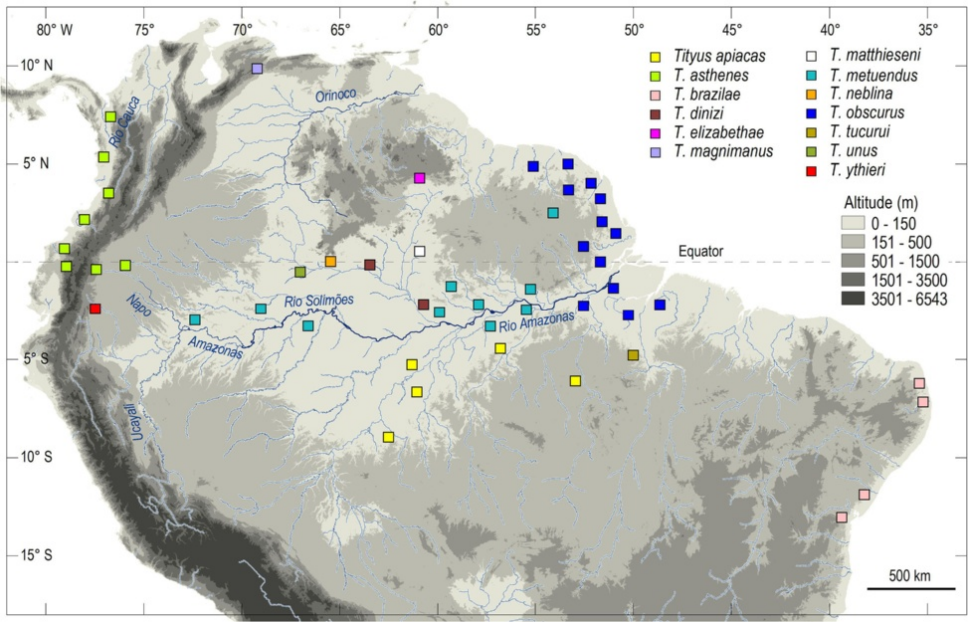
Map of northern South America – Amazonian and Guayana regions – showing the known distribution of the most important *Tityus* (*Atreus*) species

Curiously, however, the absolute number of noxious species of *Tityus* seems to remain stable. Over a period of many years, several authors have suggested that only a limited number of species can be dangerous to humans [39]. Naturally, for many years, all the publicity has gone to a small number of species, namely *T. serrulatus* and *T. bahiensis* in Brazil, and maybe to a less extend to *Tityus trinitatis* Pocock, 1897 from Trinidad and Tobago. Over the years, I have attempted to demonstrate that this number must be in fact much higher in nature. If several noxious species are totally disregarded by professionals working with public health, this has to do with the fact that these species are never in close contact with humans. In very large natural regions, such as Amazonia, human populations have been scarce for centuries. Only in recent years, they have experienced a rapid demographic expansion, with dramatic destruction of the natural environment. It is almost certain that several species of the genus *Tityus* in Amazonia, mainly belonging to the subgenus *Atreus* are highly toxic. Isolated cases of lethal incidents have been observed after stings by *Tityus metuendus* Pocock and *Tityus obscurus* Gervais [145]. Like many others, however, these are probably equilibrium species and will be partially or totally selected by the destruction of their environment before human populations become large and vulnerable to them. The number of potentially noxious *Tityus* species in Amazonia, Guayana and other rainforests of Ecuador and Peru is important and always increasing. This situation led to misidentification of species in several observed cases. Nevertheless, the regional patterns of distribution of many, if not most, of *Tityus* species in these rainforests have large regional characteristics. Consequently, very few species will be found on the north, south, western and eastern ranges of this vast region. The known patterns of distribution of several *Tityus* (subgenus *Atreus)* are illustrated in Fig. 25. Naturally, exceptions do exist, but these concern mainly polymorphic species. A few examples will be adressed in the next section.

##### Polymorphic species within the genus *Tityus*

The already discussed species *Tityus bahiensis* provides a good example of a clinal polymorphic species with a very regular gradient. This was first interpreted as a polytypic situation by Lourenço [98], with populations of species and subspecies. Such situation also illustrates the difficulties of clearly defining the biogeographic state of a given population. *T. bahiensis* is very common in the southeastern region of Brazil and represents an opportunistic species often living in unpredictable disclimax environments. Differences in the patterns of body pigmentation of individuals belonging to what appeared to be two different allopatric populations permitted the recognition of two different species [98]: *T. bahiensis* with a meridional distribution and *T. eickstedtae* Lourenço, 1982 with a septentrional distribution. Subsequently, additional data from the zone of contact between the two supposed distinct populations indicated what could be interpreted as hybrid forms. More detailed studies, however, suggested the existence of a single polytypic species with two subspecies (and two subpopulations), one in the south (*T. bahiensis*) and the second in the north (*T. bahiensis eickstedtae*) [123]. Finally, studies performed on material from additional localities showed that *T. bahiensis* is clearly a polymorphic species, as are several others in the genus *Tityus*.

*Tityus costatus* was described by Karsch [146], based on specimens collected in the state of Rio de Janeiro. This species has not an infamous reputation, although it may be responsible for a number of incidents in the coastal regions of Brazil. These incidents are certainly misidentified in several cases and attributed to *T. bahiensis* or *T. serrulatus*. Even though not much is known about the venom or toxins produced by this species, Bücherl and Pucca [147], in an already old publication, suggested that it could have a strong activity on mice.

*Tityus costatus* is an autochtonous element of the Brazilian Atlantic forest, but yet rather poorly known by non-experts. For a long time the species remained poorly characterized. Lourenço [96] stated that it was difficult to distinguish *T. costatus* from *Tityus trivittatus dorsomaculatus* Lutz & Mello. Studies of further material from several localities in the Atlantic forest, from the states of Espírito Santo to Rio Grande do Sul, established the existence of a mosaic pattern of polymorphism in *Tityus costatus* [109]. The two nominal species are only different morphs of one single species. *T. costatus* is defined as a *maculata* form and *T. dorsomaculatus* as a *trifasciata* form. The considerable variability observed for *T. costatus* concerns mainly the pigmentation patterns observed. Two major patterns are observed and related to the *maculata* and *trifasciata* forms with, however, an important gradient of intermediate forms between these two extremes. Research on the variability of differentiation patterns showed that no correlations could be drawn between latitudinal gradient and the pattern of pigmentation [109]. This type of complex pattern has previously been described by Mello [148] for a freshwater crab, *Trichodactylus fluviatilis* Latreille.

Further analysis of environmental factors and variable pigmentation patterns suggests that the *maculata* form of *T. costatus* occurs in sites ranging from sea level (very light form) to 1000 m (much darker). The *trifasciata* form was found at sites above 1000 m. The relief of this part of the Atlantic forest is very irregular; changes from sea level to more than 2000 m can occur over distances of less than 30 to 40 km. Consequently, it was suggested that *T. costatus* should be defined as a mosaic type polymorphic species whose pigmentation pattern changes with altitude. The actual environmental factor responsible for this variability seems to be the temperature. A similar example has been described in the lizard *Amphisbaena alba* [149].

The most complexes models of polymorphism observed for species of *Tityus* concerned those distributed in the Amazon and Guayana regions. Most certainly, other examples are present also for other morphoclimatical regions in South America, but remain not studied. Nevertheless, most of the well-studied populations of Amazonian scorpions are monomorphic species. Examples include *Tityus obscurus* (Gervais, 1843); *T. metuendus* Pocock, 1897; *Tityus dinizi* Lourenço, 1997; *Tityus tucurui* Lourenço, 1988; and *Tityus neblina* Lourenço, 2008. Monomorphic species either exhibit average ranges of distribution or local endemism [106, 123].

**Fig. 26:**
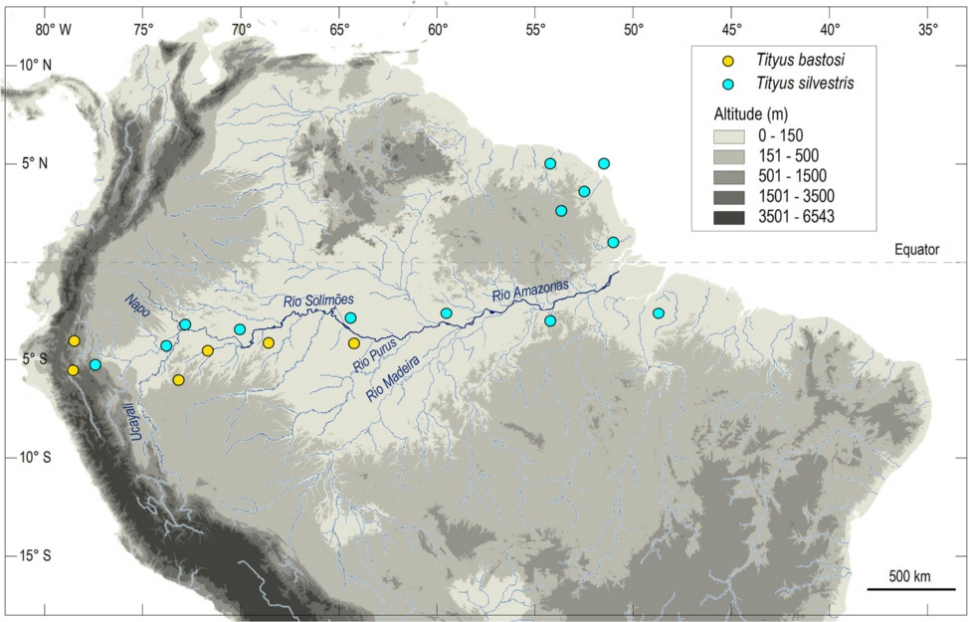
Map of northern South America – Amazonian and Guayana regions – showing the known distribution of two polymorphic species of *Tityus*

Nevertheless, analysis of different biogeographic patterns revealed at least two examples of a peculiar type of polymorphism. First, *Tityus gasci* Lourenço, 1982 shows a distribution that ranges from French Guiana to Peru and Ecuador including an important region of the forests of the Amazon and Guayana regions. Analysis of the variability of body pigmentation and the morphometrics of the chelae and metasomal segments allow the recognition of a cline along a transect from French Guiana to Ecuador. Pigmentation gradually intensifies, and the length of the chelae and metasomal segments decreases from French Guiana to Ecuador. This suggests that *T. gasci* is a clinal polymorphic species [106]. Secondly, *Tityus silvestris* Pocock, 1897 exhibits a wide distribution over the Amazon and Guayana forests. An analysis similar to that of *T. gasci* has been based on specimens from an important number of sites (Fig. 26). Pigmentation did not vary over the entire range. Morphometric values, however, displayed considerable variation that showed little geographical correlation [108, 150]. A similar pattern first encountered by botanists was defined as ‘ochlospecies’ [151].

**Fig. 27:**
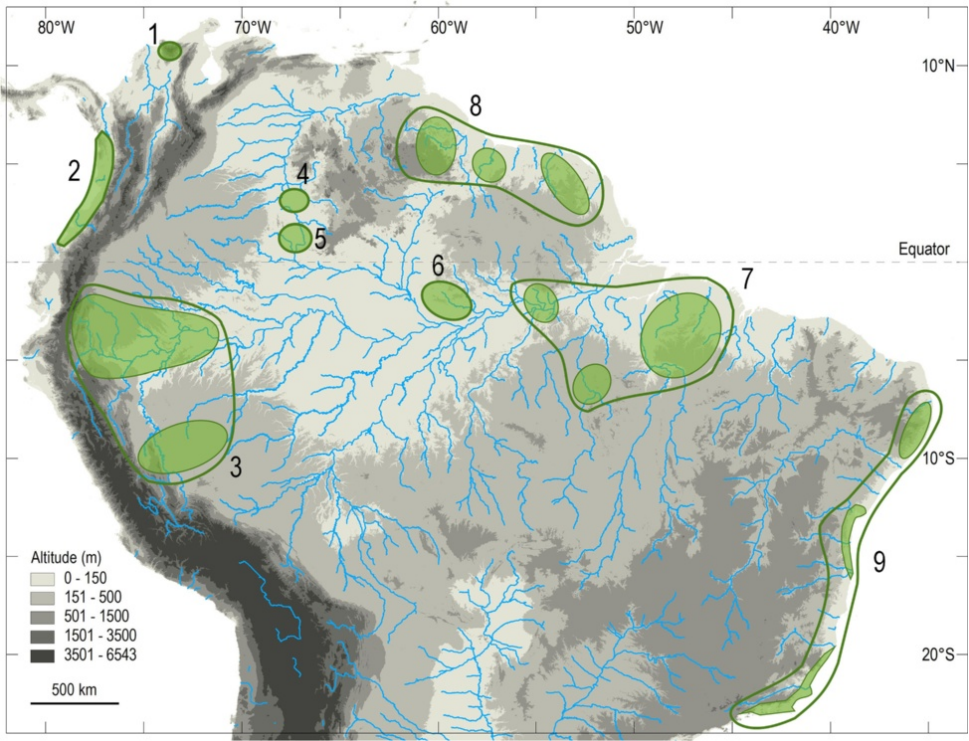
Major centers of scorpion endemism in tropical South America. For further details, refer to Lourenço [79]

According to Prance [152], ‘ochlospecies’ are common in many large genera of plants containing over 100 species. The genus *Tityus* has more than 220. As reported by Prance [152], during some of the dry periods that took place during paleoclimatic episodes, the forest cover was reduced to small patches and became fragmented into isolated allopatric populations (Fig. 27). Such isolated populations of ecologically adaptable species (what was the case with some opportunistic species of *Tityus*), rapidly re-colonized the forest that was restablished during subsequent wet episodes. Consequently, previously isolated populations became contiguous. Temporary reproductive isolation did not produce genetic incompatibility. This is apparently the case both for woody plants and for scorpions. Only minor morphological differences evolved, and when species reunited, geographical variation was no longer well correlated. Prance [152] suggested that this type of variation is attributable to recent Pleistocene climate changes, and not to actual speciation. The number of generations in both woody plants and scorpions since the last dry period, 10.000 years BP, is much less important than in other groups of organisms, such as butterflies [153]. Only a limited amount of speciation therefore probably occurred. Most valid species in all probability originated long before the recent Pleistocene paleoclimatic episodes.

**Fig. 28:**
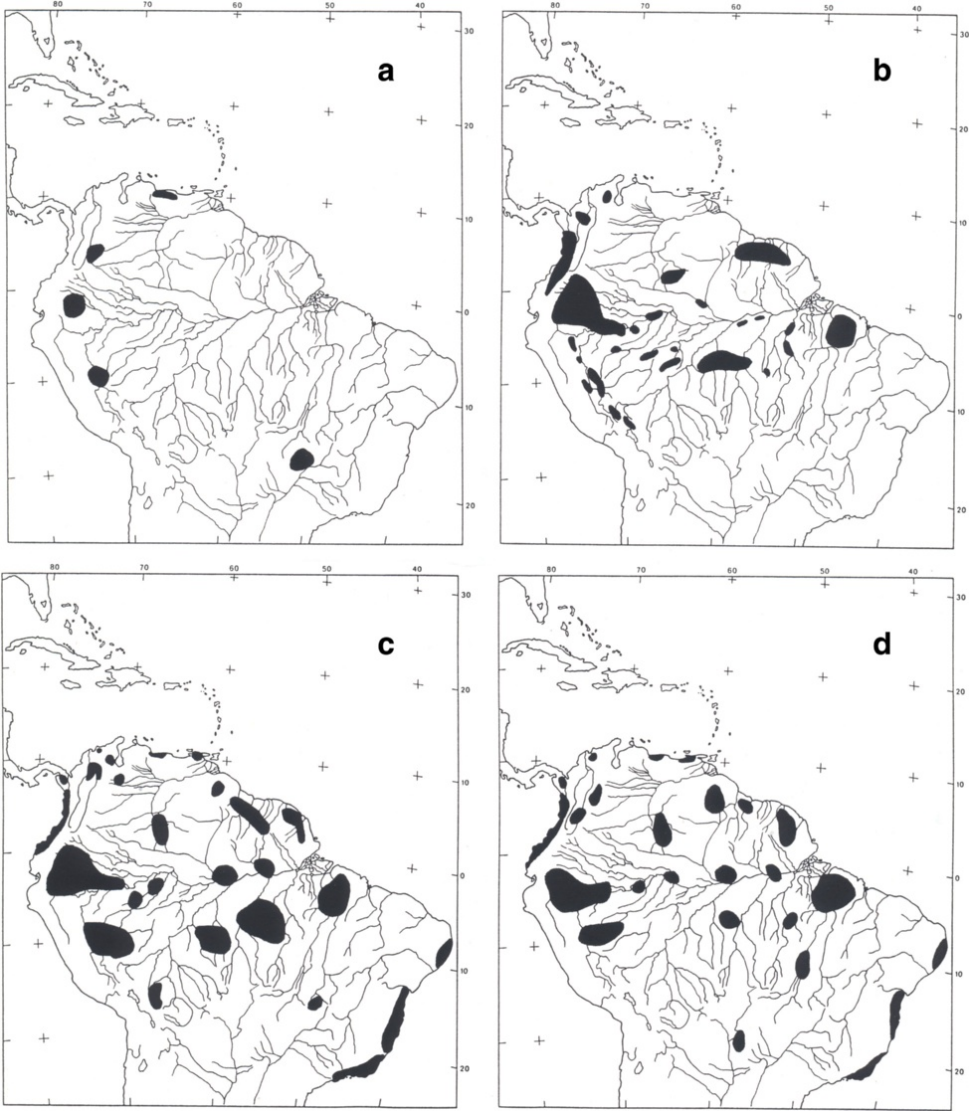
Refugia defined for several taxa in tropical South America: **a** lizards [149]; **b** birds [156, 157]; **c** woody plants [82] and **d** scorpions

In fact, the biogeographic pattern presently observed for *Tityus* species (and other scorpions from Amazon and Guayana regions) has a large connexion with all the palaeoclimatic events that took place in these regions since the end of the Cenozoic. During many decades, authors believed that biological diversity in rainforests was rich because these environments have been relatively stable for many millions of years [154, 155]. However, data from ecological, palaeoclimatic and palynological studies indicate this that ‘stability’ was interrupted by periods of climatic change during several dry/wet/dry episodes since the late Cenozoic Period and especially during the Pleistocene and Holocene epochs [82]. During the earlier Quaternary Period, temperate regions were glaciated; cooler, drier conditions prevailed in the tropical zones of today and reduced the rainforest to savannas or dry forests except in localized regions where conditions of temperature and humidity allowed the forests to persist. This historical reduction of rainforests to patch refuges is supported by existing biogeographical patterns of distribution and differentiation of several taxa [127, 152, 156–159], and by palynological and geomorphological evidence [77, 160, 161] (Figs. 27, 28).

Prance [152] postulates that the reduction of forest to small patches stimulated many changes in plant and animal populations in the refugia, ranging from extinction to an increased rate of speciation. More recent evidence for the refuge theory comes from biogeographical studies of scorpions in tropical South America [79, 106, 107]. These investigations led to the postulation of about 25 refugia or areas of high endemism (Fig. 28). The number, size and location of these refuges correlate well with those described by Prance [152] from his studies of woody plant biogeography, and studies by Haffer [156, 157] and Brown [159] on birds and butterflies, respectively, to identify regions of high endemism. Together, these studies provide a basis to identify and measure the most important centers of species diversity in South American tropical rainforests (Fig. 28).

## Conclusions

The first objective of this article was to bring general information about scorpions of medical importance, addressed to non-specialist people whose research embraces this group. The idea is to demonstrate that the group diversity and patterns of distribution are much more complex than it seems at first sight, in particular to those having access to a limited number of species. If the group diversity is important, the same should be applied to the diversity of toxins. Today, only a very limited number of distinct taxa retain the attention of toxin experts, but maybe this overview about scorpions may encourage the interest of researchers on biochemistry and molecular biology of venom toxins to expand their research to a broader array of scorpion groups, in particular for those that can be informative on the evolution of complex venoms.

It seems obvious that the taxonomy and classification of scorpions is far from being a simple task. In fact, this classification is permanently changing and this situation brings an unconfortable situation for people who use these organisms in their research. For instance, certain toxins are presently associated with species names that no longer exist in the zoological literature; one example is the ‘*Tityus cambridgei* toxin’, which has no longer a sense since the correct name for this species is now *Tityus obscurus* [162].

Nevertheless, people using scorpions in their research should be aware of more precise identifications of the species they are using in their research. In the previous sections, I exposed the quite many situations of mix-up that can be found in an important number of scientific publications. The progress observed in the taxonomy of scorpions, specially the discovery and description of a remarkable number of new species, in the last 15 to 20 created a totally new scorpiological panorama. These new descriptions concern all the known groups of scorpions, but have a conspicuous incidence on the groups presenting medical importance, such as the genera *Androctonus*, *Buthus*, *Centruroides*, *Leiurus* and *Tityus*.

In face of all these taxonomic difficulties, the best avenues should be more exchanges between people using scorpions in their research and true experts. Naturally, the number of true experts is yet very limited, but more and more young people are now being trained in scorpion taxonomy. Some previous examples of cooperation do exist in the literature and have proven their efficacity [69].

## References

[1] Jeran AJ, Brownell P, Polis GA (2001). Scorpion Biology and Research. Paleontology.

[2] Briggs DEG (1987). Scorpions take to the water. Nature.

[3] Shear WA, Kukalová-Peck J (1990). The ecology of Paleozoic terrestrial arthropods: the fossil evidence. Can J Zool.

[4] Lourenço WR, Gall JC (2004). Fossil scorpions from the Buntsandstein (Early Triassic) of France. C R Palevol.

[5] Lourenço WR, Gopalakrishnakone P (2015). Scorpion diversity and distribution; past and present patterns. Toxinology.

[6] Lourenço WR, Cuellar O (1995). Scorpions, Scorpionism, life history strategies and parthenogenesis. J Venom Anim Toxins.

[7] Lourenço WR, Cloudsley-Thompson JL, Bon C, Goyffon M (1996). Effects of human activities on the environment and the distribution of dangerous species of scorpions. Envenomings and their treatments.

[8] Lourenço WR, Knox MB, Yoshizawa AC (1994). L’invasion d’une communauté à le stade initial d’une succession secondaire par une espèce parthénogénétique de Scorpion. Biogeographica.

[9] Lourenço WR, Cloudsley-Thompson JL, Cuellar O, von Eickstedt VRD, Barraviera B, Knox MB (1996). The evolution of scorpionism in Brazil in recent years. J Venom Anim Toxins.

[10] Lourenço WR (2002). Scorpions of Brazil.

[11] Lourenço WR, Adis J (2002). Scorpions. Amazonian Arachnida and Myriapoda.

[12] Lourenço WR, Cloudsley-Thompson JL (1996). The evolutionary significance of colour, colour patterns and fluorescence in scorpions. Rev suisse Zool..

[13] Adis J, Adis J, Adis J (2002). Recommended sampling techniques. Amazonian Arachnida and Myriapoda.

[14] Lourenço WR, Toft S, Scharff N (2002). Reproduction in scorpions, with special reference to parthenogenesis. European Arachnology 2000.

[15] Leach WE (1815). A tabular view of the external characters of four classes of animals, which Linné arranged under Insecta; with the distribution of the genera composing three of these classes into orders, etc. and descriptions of several new genera and species. Transactions of the Linnean Society of London.

[16] Vachon M (1952). Etudes sur les Scorpions.

[17] Lourenço WR (2003). Compléments à la faune de scorpions (Arachnida) de l’Afrique du Nord, avec des considérations sur le genre *Buthus* Leach, 1815. Rev suisse Zool.

[18] Lourenço WR (2005). Description of a new scorpion species of the genus *Buthus* Leach, 1815 (Scorpiones, Buthidae) from Guinea and Senegal in Western Africa. Entomol Mitt Zool Mus Hamb.

[19] Lourenço WR (2005). A new species of the genus *Buthus* Leach, 1815 (Scorpiones, Buthidae) from Senegal and Niger in Western Africa. Entomol Mitt Zool Mus Hamb.

[20] Lourenço WR, Geniez P (2005). A new scorpion species of the genus *Buthus* Leach, 1815 (Scorpiones, Buthidae) from Morocco. Euscorpius.

[21] Lourenço WR (2015). Deux nouvelles espèces de scorpions de la famille des Buthidae C. L. Koch, 1837 collectées dans le Parc National de Zakouma au Tchad. Rev Iber Aracnol..

[22] Sadine SE, Bissati S, Lourenço WR (2016). The first true deserticolous species of *Buthus* Leach, 1815 from Algeria (Scorpiones, Buthidae); ecological and biogeographic considerations. C R Biol.

[23] Billen B, Tytgat J, Lima ME (2009). The venom of the Asian scorpion *Buthus martensi* Karsch. An overview of the toxins and their biological targets. Animal toxins: State of the Art.

[24] Hemprich FW, Ehrenberg CG. Zoologica II. Arachnoidea. Plate I: *Buthus*; plate II: *Androctonus*. In: Symbolae Physicae seu Icones et Descriptiones Animalium evertebratorum sepositis Insectis quae ex itinere per Africam borealem et Asiam occidentalem. Friderici Guilelmi Hemprich et Christiani Godofredi Ehrenberg, medicinae et chirurgiae doctorum, studio novae aut illustratae redierunt. Percensuit et regis iussu et impensis edidit Dr. C. G. Ehrenberg. Decas prima. Berlin: Berolini ex officina Academica, Venditur a Mittlero, 1828.

[25] Lourenço WR (2005). Nouvelles considérations taxonomiques sur les espèces du genre *Androctonus* Ehrenberg, 1828 et description de deux nouvelles espèces (Scorpiones, Buthidae). Rev suisse Zool.

[26] Levy G, Amitai P (1980). Fauna Palaestina, Arachnida I: Scorpiones.

[27] Lourenço WR (2008). A new species of *Androctonus* Ehrenberg, 1828 from Togo (Scorpiones, Buthidae). Entomol Mitt Zool Mus Hamb.

[28] Lourenço WR (2015). A new species of *Androctonus* Ehrenberg, 1828 from the Aïr Massif in Niger (Scorpiones: Buthidae). Serket.

[29] Lourenço WR, Qi JX (2006). A new species of *Androctonus* Ehrenberg, 1828 from Afghanistan (Scorpiones, Buthidae). Zool Middle East.

[30] Lourenço WR, Qi JX (2007). A new species of *Androctonus* Ehrenberg, 1828 from Mauritania (Scorpiones, Buthidae). Bol SEA..

[31] Lourenço WR, Ythier E, Leguin EA (2009). A new species of *Androctonus* Ehrenberg, 1828 from Morocco (Scorpiones: Buthidae). Euscorpius..

[32] Lourenço WR, Duhem B, Cloudsley-Thompson JL (2012). Scorpions from Ennedi, Kapka and Tibesti the mountains of Chad, with descriptions of nine new species (Scorpiones: Buthidae, Scorpionidae). Arthropoda Sel.

[33] Lourenço WR, Rossi A, Sadine SE (2015). More about the genus *Androctonus* Ehrenberg, 1828 (Scorpiones, Buthidae), with the description of a new species from Ethiopia. Arachnida..

[34] Lourenço WR, Leguin EA (2015). A new species of *Compsobuthus* Vachon, 1949 (Scorpiones: Buthidae) from Armenia. Zool Middle East.

[35] Cloudsley-Thompson JL (1961). Observations on the biology of the scorpion, *Leiurus quinquestriatus* (H & E) in the Sudan. Entomol Mon Mag..

[36] Cloudsley-Thompson JL (1963). Some aspects of the physiology of *Buthotus minax* (Scorpiones: Buthidae) with remarks on other African scorpions. Entomol Mon Mag..

[37] Levy G, Amitai P, Shulov A (1970). *Leiurus quinquestriatus hebraeus* (Birula, 1908) (Scorpiones, Buthidae) and its systematic position. Israel J Zool.

[38] Cloudsley-Thompson JL (1992). Scorpions. Biologist.

[39] Simard JM, Watt DD, Polis G, Polis GA (1990). Venoms and Toxins. The Biology of Scorpions.

[40] Loret E, Hammock B, Brownell P, Polis GA (2001). Structure and Neurotoxicity of Venoms. Scorpion Biology and Research.

[41] Cloudsley-Thompson JL (1962). Lethal temperatures of some desert arthropods and the mechanism of heat death. Entomologia Experimentalis et Applicata..

[42] Kraepelin K (1891). Revision der Skorpione. I. Die Familie der Androctonidae. Jahrb Hamburg Wiss Anst.

[43] Vachon M (1949). Etudes sur les Scorpions. III (suite). Description des Scorpions du Nord de l’Afrique. Arch Inst Pasteur Alger.

[44] Vachon M (1979). Arachnids of Saudi Arabia. Scorpiones. Fauna Saudi Arabia..

[45] Sissom WD (1994). Descriptions of new and poorly known scorpions of Yemen (Scorpiones: Buthidae, Diplocentridae, Scorpionidae). Fauna Saudi Arabia..

[46] Lourenço WR, Modry D, Amr Z (2002). Description of a new species of *Leiurus* Ehrenberg 1828 (Scorpiones, Buthidae) from the South of Jordan. Rev suisse Zool.

[47] Hendrixson BE (2006). Buthid scorpions of Saudi Arabia, with notes on other families (Scorpiones: Buthidae, Liochelidae, Scorpionidae). Fauna of Arabia..

[48] Lourenço WR, Qi JX, Cloudsley-Thompson JL (2006). The African species of the genus *Leiurus* Ehrenberg, 1828 (Scorpiones: Buthidae) with the description of a new species. Bol SEA..

[49] Lowe G, Yagmur EA, Kovarik F (2014). A review of the genus *Leiurus* Ehrenberg, 1828 (Scorpiones: Buthidae) with description of four new species from the Arabian Peninsula. Euscorpius..

[50] Lourenço WR, Rossi A (2016). One more African species of the genus *Leiurus* Ehrenberg, 1828 (Scorpiones: Buthidae) from Somalia. Arachnida..

[51] Birula AA (1908). Ergebnisse der mit Subvention aus der Erbschaft Treitl unternommenen zoologischen Forschungsreise Dr. F. Werner’s nach dem Anglo-Aegyptischen Sudan und Nord-Uganda. XIV. Skorpiones und Solifugae. Sitzungsberichte der kaiserlich-königlichen Akademie der Wissenchaften, Wien.

[52] Francke OF (1985). Conspectus genericus scorpionorum 1758–1982 (Arachnida: Scorpiones). Occas Pap Mus, Texas Tech University..

[53] Lourenço WR (2008). About the presence of the genus *Buthus* Leach, 1815 in the Arabian Peninsula and description of a new species (Scorpiones, Buthidae). Entomol Mitt Zool Mus Hamb.

[54] Linnaeus C (1758). Systema Naturae per regna tria Naturae, secundum Classes, Ordines, Genera, Species, cum Characteribus, Differentiis, Synonymis, Locis. Ed. 10. Laurentii Salvii, Holmiae (Stockholm).

[55] Zlotkin E, Miranda F, Rochat H, Bettini S (1978). Venoms of Buthinae. C. Chemistry and pharmacology of Buthinae scorpion venoms. Arthropod Venoms.

[56] Lourenço WR (2009). Reanalaysis of the genus *Scorpio* Linnaeus 1758 in Sub-Saharan Africa and description of one new species from Cameroon (Scorpiones, Scorpionidae). Entomol Mitt Zool Mus Hamb.

[57] Lourenço WR, Cloudsley-Thompson JL (2009). A new species of the genus *Scorpio* Linnaeus 1758 from Sudan (Scorpiones, Scorpionidae). Bol SEA..

[58] Lourenço WR, Cloudsley-Thompson JL (2012). About the enigmatic presence of the genus *Scorpio* Linnaeus, 1758 in Congo with the description of a new species from Niger (Scorpiones, Scorpionidae). Serket.

[59] Lourenço WR, Rossi A (2016). Confirmation of a new species of *Scorpio* Linnaeus, 1758 in the Tassili N’Ajjer, Mountains, South Algeria (Scorpiones: Scorpionidae). Onychium..

[60] Talal S, Tesler I, Sivan J, Ben-Shlomo R, Tahir HM, Prendini L (2015). Scorpion speciation in the Holy Land: Multilocus phylogeography corroborates diagnostic differences in morphology and burrowing behavior among *Scorpio* subspecies and justified recognition as phylogenetic, ecological and biological species. Mol Phylogenet Evol.

[61] Vachon M (1958). Scorpions, Mission scientifique au Tassili des Ajjer (1949). Travaux de l’Institut de recherches sahariennes de l’Université d’Alger. Zoologie.

[62] Monod L, Lourenço WR (2005). Hemiscorpiidae (Scorpiones) from Iran, with descriptions of two new species and notes on biogeography and phylogenetic relationships. Rev suisse Zool.

[63] Lowe G (2010). Two new *Hemiscorpius* Peters, 1861 (Scorpiones: Hemiscorpiidae) from Northern Oman. Euscorpius..

[64] Radmanesh M (1990). Clinical study of *Hemiscorpius lepturus* in Iran. J Trop Med Hyg.

[65] Radmanesh M (1998). Cutaneous manifestations of the *Hemiscorpius lepturus* sting: a clinical study. Int J Dermatol..

[66] Lourenço WR (2011). The genus *Hemiscorpius* Peters, 1861 (Scorpiones: Hemiscorpiidae) in East Africa, and description of a new species from Somalia. Entomol Mitt Zool Mus Hamburg.

[67] Lourenço WR (2011). More about the African species of *Hemiscorpius* Peters, 1861 (Scorpiones: Hemiscorpiidae), and a description of a new species from Egypt. Bol SEA..

[68] Lourenço WR, Sissom WD, Bousquets JL, Gonzalez Soriano E, Papavero N (2000). Scorpiones. Biodiversidad taxonomia y biogeografia de artropodos de México: Hacia una sintesis de su conocimiento II.

[69] Fuentes-Silva D, Santos-Jr AP, Oliveira JS (2014). Envenomation caused by *Rhopalurus amazonicus* Lourenço, 1986 (Scorpiones, Buthidae) in Pará state, Brazil. J Venom Anim Toxins incl Trop Dis..

[70] Lourenço WR (2008). The geographic pattern of distribution of the genus *Rhopalurus* Thorell, 1876 in the Guayana-Amazon region (Scorpiones: Buthidae). Euscorpius..

[71] Mori SA (1991). The Guayana lowland floristic Province. Biogeographica.

[72] Lourenço WR (1986). Biogéographie et phylogenie des scorpions du genre *Rhopalurus* (Scorpiones, Buthidae). Mém Soc R Belge Ent..

[73] Lourenço WR, Sastre C (1988). Les savanes néotropicales; caractéristiques écologiques et biogéographiques. Exemples de modalités de peuplement chez les scorpions et chez les phanérogames. Biogeographica.

[74] Teruel R, Roncallo CA (2008). Rare or poorly known scorpions from Colombia. III. On the taxonomy and distribution of *Rhopalurus laticauda* Thorell, 1876 (Scorpiones: Buthidae), with description of a new species of the genus. Euscorpius.

[75] Teruel R (2006). Apuntes sobre la taxonomía y biogeografía del género *Rhopalurus* Thorell 1876 (Scorpiones: Buthidae), con la descripción de dos nuevas especies de Cuba. Bol SEA..

[76] Teruel R, Tietz AK (2008). The true identity of *Rhopalurus pintoi* Mello-Leitão, 1932, with notes on the status and distribution of *Rhopalurus crassicauda* Caporiacco, 1947 (Scorpiones: Buthidae). Euscorpius..

[77] Ab’Saber AN (1977). Espaços ocupados pela expansão dos climas secos na América do Sul, por ocasião dos períodos glaciais quaternários. Paleoclimas..

[78] Van der Hammen T, Bourlière F (1983). The palaeoecology and palaeogeography of savannas. Tropical Savannas.

[79] Lourenço WR, Brownell P, Polis GA (2001). Scorpion diversity in Tropical South America: Implications for conservation programs. Scorpion biology and research.

[80] Murça Pires J, Prance GT, Prance GT, Lovejoy TE (1985). The vegetation types of the Brazilian Amazon. Amazonia.

[81] Lourenço WR (1982). Révision de genre *Rhopalurus* Thorell, 1876 (Scorpiones, Buthidae). Rev Arachnol..

[82] Prance GT editor. Biological diversification in the tropics. Columbia Univ. Press, New York: 1982. 714 p.

[83] Mayr E (1931). Notes on *Halcyon chloris* and some of its subspecies. Am Mus Novit..

[84] Bernardi G. Les catégories taxonomiques de la systématique évolutive. Les problèmes de l’espèce dans le règne animal. Soc Zool Fr. 1980:373–425

[85] Birula AA (1910). Ueber *Scorpio maurus* Linné und seine Unterarten. Horae Societatis Entomologicae Rossicae..

[86] Lourenço WR (2006). Nouvelle proposition de découpage sous-générique du genre *Tityus* C.L. Koch, 1836 (Scorpiones, Buthidae). Bol SEA.

[87] Lourenço WR (2015). What do we know about some of the most conspicuous scorpion species of the genus *Tityus*? A historical approach. J Venom Anim Toxins incl Trop Dis.

[88] Koch CL (1836). *Die Arachniden*. CH Zeh’sche Buchhandlung, Nürnberg.

[89] Perty M. *Delectus animalium articulatorum quae in itinere per Brasiliam. Collegerum* JB de Spix, CF Ph de Martius editor’s. Hamburg-Londres: 1833 (1834). 200 p.

[90] Kraepelin K, Dahl F (1899). Scorpiones und Pedipalpi. Das Tierreich.

[91] Kraepelin K (1910). Neue Beiträge zur Systematik der Gliederspinnen. Mitteilungen aus dem Naturhistorischen Museum 2. Beiheft zum Jahrbuch der Hamburgischen wissenschaftlichen Anstalten.

[92] Mello-Campos O (1924). Os escorpiões Brazileiros. Mem Inst Oswaldo Cruz.

[93] Mello-Leitão C (1931). Divisão e distribuição do genero *Tityus* Koch. An Acad Bras Cienc.

[94] Mello-Leitão C (1939). Revisão do genero *Tityus*. Physis..

[95] Mello-Leitão C (1945). Escorpiões sul-americanos. Arq Mus Nac (Rio de J.).

[96] Lourenço WR (1980). Contribution à la connaissance systématique des scorpions appartenant au complexe *Tityus trivittatus* Kraepelin, 1898 (Buthidae). Bull Mus Nat His Nat, Paris.

[97] Lourenço WR (1981). Sur la systématique des scorpions appartenant au complexe *Tityus stigmurus* (Thorell, 1877) (Buthidae). Rev Bras Biol.

[98] Lourenço WR (1982). La véritable identité de *Tityus bahiensis* (Perty, 1834). Description de *Tityus eickstedtae* n. sp. (Scorpiones, Buthidae). Rev Arachnol.

[99] Lourenço WR (1984). Analyse taxonomique des scorpions du groupe *Tityus clathratus* Koch, 1845 (Scorpiones, Buthidae). Bull Mus Nat Hist Nat, Paris..

[100] Lourenço WR (1984). Etude systématique de quelques espèces appartenant au complexe *Tityus forcipula* (Gervais, 1844) (Scorpiones, Buthidae). Bull Mus Nat His Nat, Paris..

[101] Lourenço WR, Maury EA (1985). Contribution à la connaissance systématique des scorpions appartenant au ‘complexe’ *Tityus bolivianus* Kraepelin, 1895 (Scorpiones, Buthidae). Rev Arachnol.

[102] Lourenço WR, Eickstedt VRD (1987). Contribuição ao conhecimento taxonômico dos escorpiões associados ao grupo *Tityus melanostictus* Pocock, 1893 (Scorpiones, Buthidae). Mem Inst Butantan.

[103] Lourenço WR (1988). La faune des scorpions de l’Equateur. I. Les Buthidae. systématique et biogéographie. Rev Suisse Zool.

[104] Lourenço WR (1994). Scorpions (Chelicerata) de Colombie. VI. Quatre nouvelles espèces de Buthidae des régions Amazonienne, Sud-Pacifique et de la Cordillère Orientale. Rev Acad Colombiana Cienc Exat Fís Natur.

[105] Lourenço WR (2000). Synopsis of the Colombian species of *Tityus* Koch (Chelicerata, Scorpiones, Buthidae), with descriptions of three new species. J Nat Hist.

[106] Lourenço WR (1986). Diversité de la faune scorpionique de la région amazonienne; centres d’endémisme; nouvel appui a la théorie des refuges forestiers du Pléistocène. Amazoniana.

[107] Lourenço WR (1987). Les modèles évolutifs des scorpions néotropicaux et la théorie des refuges forestiers du Pléistocène. C R Soc Biogéogr.

[108] Lourenço WR (1988). Diversité biologique et modalités de la spéciation chez les scorpions amazoniens; *Tityus silvestris* Pocock, un cas particulier de polymorphisme. C R Acad Sci, Paris..

[109] Lourenço WR, Eickstedt VRD (1988). Considerações sobre a sistemática de *Tityus costatus* (Karsch, 1879), provável espécie polimórfica de escorpião da Floresta Atlântica do Brasil (Scorpiones, Buthidae). Iheringia Sér Zool..

[110] González-Sponga MA (1996). Guía para identificar escorpiones de Venezuela.

[111] Lourenço WR (2003). Description of a new species of *Tityus* (Scorpiones, Buthidae) from Serra da Jurema in the state of Bahia, Brazil. Rev Iber Aracnol..

[112] Lourenço WR (2005). Scorpion diversity and endemism in the Rio Negro region of Brazilian Amazonia, with the description of two new species of *Tityus* C. L. Koch (Scorpiones, Buthidae). Amazoniana.

[113] Lourenço WR (2012). Further considerations on *Tityus* (*Archaeotityus*) *clathratus* C. L. Koch, 1844 and description of two associated new species (Scorpiones, Buthidae). Bol Soc Entomol Arag.

[114] Lourenço WR (2013). A new species of *Tityus* C. L. Koch (Scorpiones, Buthidae) from the Island of Martinique, Lesser Antilles. Arthrop Sel.

[115] Lourenço WR, Ythier E (2013). The remarkable scorpion diversity in the Ecuadorian Andes and description of a new species of *Tityus* C. L. Koch, 1836 (Scorpiones, Buthidae). ZooKeys.

[116] Lourenço WR (1999). Origines et affinités des scorpions des Grandes Antilles: Le cas particulier des éléments de la famille des Buthidae. Biogeographica.

[117] Armas LF, Abud-Antun AJ (2004). Adiciones al género *Tityus* C. L. Koch, 1836 en República Dominicana, con la descripción de dos especies nuevas (Scorpiones : Buthidae). Rev Ibér Aracnol.

[118] Lourenço WR, Pézier A (2002). Addition to the scorpion fauna of the Manaus region (Brazil), with a description of two new species of *Tityus* from the canopy. Amazoniana.

[119] Polis GA, Polis GA (1990). Ecology. The Biology of Scorpions.

[120] Pianka ER (1988). Evolutionary Ecology.

[121] Haffer J, Nelson G, Rosen DE (1981). Aspects of Neotropical bird speciation during the Cenozoic. Vicariance biogeography: a critique.

[122] Eiten G (1974). An outline of the vegetation of South America.

[123] Lourenço WR (1986). Les modèles de distribution géographique de quelques groupes de scorpions néotropicaux. C R Soc Biogéogr.

[124] Goodnight J, Goodnight ML (1953). The Opilionid faune of Chiapas Mexico, and adjacent areas (Arachnoidea, Opiliones). Am Mus Novit..

[125] Lourenço WR (1995). Les scorpions (Chelicerata, Scorpiones) de l’Equateur, avec quelques considérations sur la biogéographie et la diversité des espèces. Rev suisse Zool.

[126] Lourenço WR (1990). Caractéristiques biogéographiques de la Caatinga brésilienne. Associations avec le Chaco et d’autres formations végétales ouvertes de l’Amérique du Sud. L’exemple des scorpions. C R Soc Biogéogr.

[127] Prance GT (1973). Phytogeographic support for the theory of Pleistocene forest refuges in the Amazon Basin, based on evidence from distribution patterns in Caryocaraceae, Chrysobalanaceae, Dichapetalaceae and Lecythidaceae. Acta Amaz.

[128] Frailey CD, Lavina EL, Rancy A, Souza Filho JP (1988). A proposed Pleistocene/Holocene lake in the Amazon basin and its significance to Amazonian geology and biogeography. Acta Amaz.

[129] Lourenço WR (1979). La biologie sexuelle et le développement post-embryonnaire du scorpion Buthidae: *Tityus trivittatus fasciolatus* Pessôa, 1935. Rev Nordestina Biol.

[130] Lourenço WR (1981). Sur l’écologie du scorpion Buthidae: *Tityus trivittatus fasciolatus* Pessôa, 1935. Vie Milieu.

[131] Lourenço WR (1995). *Tityus fasciolatus* Pessôa, scorpion Buthidae à traits caractéristiques d'une espèce non-opportuniste. Biogeographica.

[132] Mathews AGA (1977). Studies on Termites from the Mato Grosso State, Brazil.

[133] Lutz A, Mello O (1922). Cinco novos escorpiões dos gêneros *Tityus* e *Rhopalurus*. Fol Méd.

[134] Matthiesen FA (1962). Parthenogenesis in scorpions. Evolution.

[135] Lourenço WR (2008). Parthenogenesis in scorpions: Some history – new data. J Venom Anim Toxins incl Trop Dis.

[136] Lourenço WR, Cloudsley-Thompson JL (1999). Discovery of the sexual population of *Tityus serrulatus*, *alias* the ‘confluenciata’ form within the complex ‘*Tityus stigmurus*’ (Thorell) (Scorpiones, Buthidae). J Arachnol.

[137] De Souza CAR, Candido DM, Lucas SM, Brescovit AD (2009). On the *Tityus stigmurus* complex (Scorpiones, Buthidae). Zootaxa..

[138] Vandel A (1928). La parthénogenèse géographique: Contribution à l’étude biologique et cytologique de la parthénogenèse naturelle. Bull Biol Fr Belg..

[139] Udvardy MDF (1969). Dynamic zoogeography. With special reference to land animals.

[140] Cuellar O (1977). Animal parthenogenesis. Science..

[141] Cuellar O (1994). Biogeography of parthenogenetic animals. Biogeographica.

[142] Glesener RR, Tilman D (1978). Sexuality and the components of environmental uncertainty: clues from geographic parthenogenesis in terrestrial animals. Amer Nat..

[143] Blondel J (1976). Stratégies démographiques et successions écologiques. Bull Soc Zool Fr..

[144] Hubbell TH, Norton RM (1978). The systematics and Biology of the cave crickets of the North American tribe Hadenoecini (Orthoptera), Saltatoria, Ensifera, Rhaphidophoridae, Dolichopodinae. Miscilaneus Publications. Mus Zool Mich..

[145] Lourenço WR (2011). The distribution of noxious species of scorpions in Brazilian Amazonia: the genus *Tityus* C. L. Koch, 1836, subgenus *Atreus* Gervais, 1843 (Scorpiones, Buthidae). Entomol Mitt Zool Mus Hamburg.

[146] Karsch F (1879). Scorpionologische Beiträge. Part II Mitt Münch Entomol Ver..

[147] Bücherl W, Pucca N (1956). Escorpiões e escorpionismo no Brasil. III. Titulação por meio de camundongos das peçonhas de *Tityus costatus* (Karsch), *Tityus trivittatus* Kraepelin, 1898 e *Bothriurus bonariensis* (Koch), 1842. Mem Inst Butantan.

[148] Mello GAS (1967). Diferenciação geografica e dimorfismo sexual de *Trichodactylus* (*Trichodactylus*) *fluviatilis* Latreille, 1825 (Crustacea, Brachyura). Pap Avulsos Zool (São Paulo).

[149] Vanzolini PE. Zoologia sistemática, geografia e a origem das espécies. Série Teses e Monografias, 3, IGEOG-USP: 1970. p. 1–56.

[150] Lourenço WR (2016). A propos de quelques amendements sur quelques espèces du genre *Tityus* C. L. Koch, 1836 (Scorpiones: Buthidae) de la région amazonienne. Arachnida.

[151] White F (1962). Geographic variation and speciation in Africa with particular reference to *Diospyros*. Syst Assoc Publ..

[152] Prance GT, Prance GT (1982). Forest refuges: Evidence from woody angiosperms. Biological diversification in the tropics.

[153] Turner JRG (1971). Two thousand generations of hybridization in a *Heliconius* butterfly. Evolution..

[154] Federov AA (1966). The structure of the tropical rain forest and speciation in the humid tropics. J Ecol..

[155] Richards PW (1969). Speciation in the tropical rain forest and the concept of the niche. Biol J Linnaeus Soc..

[156] Haffer J (1969). Speciation in Amazonian forest birds. Science..

[157] Haffer J (1974). Avian speciation in Tropical South America.

[158] Vanzolini PE, Williams EE (1970). South American Anoles: geographic differentiation and the evolution of the *Anolis chrysolepis* species group (Sauria, Iguanidae). Arq Zool (São Paulo).

[159] Brown KS, Prance GT (1982). Paleoecology and Regional Patterns of Evolution in Neotropical Forest Butterflies. Biological Diversification in the Tropics.

[160] Van der Hammen T (1974). The Pleistocene changes of vegetation and climate in Tropical South America. J Biogeogr..

[161] Van der Hammen T, Prance GT (1982). Paleoecology of Tropical South America. Biological Diversification in the Tropics.

[162] Lourenço WR, Leguin EA (2008). The true identity of *Scorpio* (*Atreus*) *obscurus* Gervais, 1843 (Scorpiones, Buthidae). Euscorpius..

